# The vicious cycle of the public's irrational use of antibiotics for upper respiratory tract infections: A mixed methods systematic review

**DOI:** 10.3389/fpubh.2022.985188

**Published:** 2022-10-20

**Authors:** Lixia Duan, Chaojie Liu, Dan Wang, Rujiao Lin, Pan Qian, Xinping Zhang, Chenxi Liu

**Affiliations:** ^1^School of Medicine and Health Management, Tongji Medical School, Huazhong University of Science and Technology, Wuhan, China; ^2^School of Psychology and Public Health, La Trobe University, Melbourne, VIC, Australia

**Keywords:** COM-B, CBM, upper respiratory tract infections, the public, antibiotic, systematic review

## Abstract

**Background:**

The public's irrational use of antibiotics for upper respiratory tract infections (URTIs) is prevalent worldwide. This study aims to synthesize evidence on how people use antibiotics to treat URTIs, its prevalence and determinants.

**Methods:**

A mixed methods systematic review was conducted using a convergent segregated approach. Relevant studies were searched from PubMed, Cochrane Library, Embase, and Web of Science. A qualitative analysis was initiated, exploring the public's antibiotic use experience for URTIS based on the Consumer Behavior Model (CBM). This was followed by a quantitative synthesis, tapping into the prevalence and predictors of public behavior in antibiotic usage for URTIs. The segregated syntheses complemented each other and were further integrated.

**Results:**

A total of 86 studies were included: 48 quantitative, 30 qualitative, eight mixed methods studies. The included studies were conducted in Europe (*n* = 29), Asia (*n* = 27) and North America (*n* = 21), assessing the behaviors of patients (*n* = 46), their parents or caregivers (*n* = 31), or both (*n* = 9). Eleven themes emerged covering the six CBM stages: need recognition, information searching, alternative evaluation, antibiotic obtaining, antibiotic consumption, and post-consumption evaluation. The six stages reinforce each other, forming a vicious cycle. The high prevalence of the public's irrational use of antibiotics for URTIs is evident despite the high heterogeneity of the studies (ranging from 0.0 to 92.7%). The perceived seriousness of illness and misbelief in antibiotics were identified consistently across the studies as the major motivation driving the public's irrational use of antibiotics for URTIs. However, individual capacity (e.g., knowledge) and opportunity (e.g., contextual restriction) in reducing antibiotic use have mixed effect.

**Conclusion:**

Systemic interventions concerning both supply and demand sides are warranted. The public needs to be educated about the appropriate management of URTIs and health care providers need to re-shape public attitudes toward antibiotic use for URTIs through communication and prescribing practices.

**Systematic review registration:**

https://www.crd.york.ac.uk/prospero, identifier: CRD42021266407.

## Background

Antibiotic resistance (AR) is one of the most concerning threats to public health and economic development recognized by people all over the world (1–3). Without effective counter-measures, AR is projected to result in an annual loss of 10 million lives worldwide by the year 2050, becoming a leading cause of death (1). Although the development of AR is a natural phenomenon, the overuse of antibiotics can fuel its occurrence (4). Thus, the responsible use of antibiotics is critical (5), especially when the emergence of AR outpaces the development of novel antibiotics (6).

The general public plays an important role in the control of antibiotic use (7). Existing studies have revealed that people often purchase antibiotics without a prescription and use antibiotics to self-treat minor illnesses for themselves and those under their care (8). The high level of antibiotic usage also induces unnecessary expectations from the public (9), which puts healthcare providers under great pressure (10), driving the over-prescription of antibiotics (10, 11). Meanwhile, non-adherence to prescribed antibiotic therapy is prevalent worldwide (12–14), further accelerating the emergence of AR. A recent study from China shows that self-actions of the public driven by irrational expectations have surpassed irrational antibiotic prescribing, becoming the leading cause of the misuse of antibiotics for URTIs (15).

Extensive studies have been conducted to explore the knowledge, attitudes and behaviors of the public in relation to antibiotic consumption under various socioeconomic circumstances (12, 16–19). Many interventions, both individual-based and population-based, have been designed to improve the antibiotic knowledge of the public using traditional (e.g., printed leaflets) and modern (e.g., social media) communication channels (20, 21). Unfortunately, these interventions have had a limited effect in most settings (20, 21). A recent study conducted in the UK suggests that a high awareness of AR may even exacerbate the overuse of antibiotics as people fear the possibility of missing out the potential protection offered by antibiotics (22). Interestingly, many countries have witnessed a dramatic decrease in antibiotic consumption during the COVID-19 pandemic (23, 24). This coincides with the increased threshold for care-seeking and fewer consultations in primary care for URTIs caused by restrictive measures, consumer hesitancy, strict infection control protocol, or a combination of all these factors.

URTIs are the predominant condition that contributes to the irrational use of antibiotics. Given that most URTIs are minor and self-limiting, it is desirable to reduce unnecessary care-seeking for URTIs (23, 24). However, this is easier to say than do. Ensuring universal access to healthcare has been a daunting task in many health care systems. It does not make sense to create access barriers. Obviously, the onus rests with the public. Patient understanding of diseases and treatment (25, 26) is not always aligned with that of health professionals (27). They often hold certain expectations through self-assessment prior to medical consultations (28). It is not unusual for health care providers to complain about the difficulty of changing the mind of their patients (10, 11). Patients may also engage in doctor shopping to satisfy their demands. In some systems, consumers can even obtain antibiotics without a prescription. Researchers have therefore called for a better targeted and more effective approach to public interventions based on a comprehensive understanding of the whole process of antibiotic consumer behaviors (20, 21), with a hope that this will help inform strategies for maintaining the already reducing antibiotic consumption during the COVID-19 pandemic (23, 24).

This mixed methods systematic review responds to the aforementioned call, aiming at answering the following questions: (1) How do people decide to use or not to use antibiotics for URTIs? (2) How prevalent is the irrational use of antibiotics for URTIs and which factors contribute to the decision of consumers in regard to the use of antibiotics for URTIs?

## Methods

### Study design

**Figure 1:**
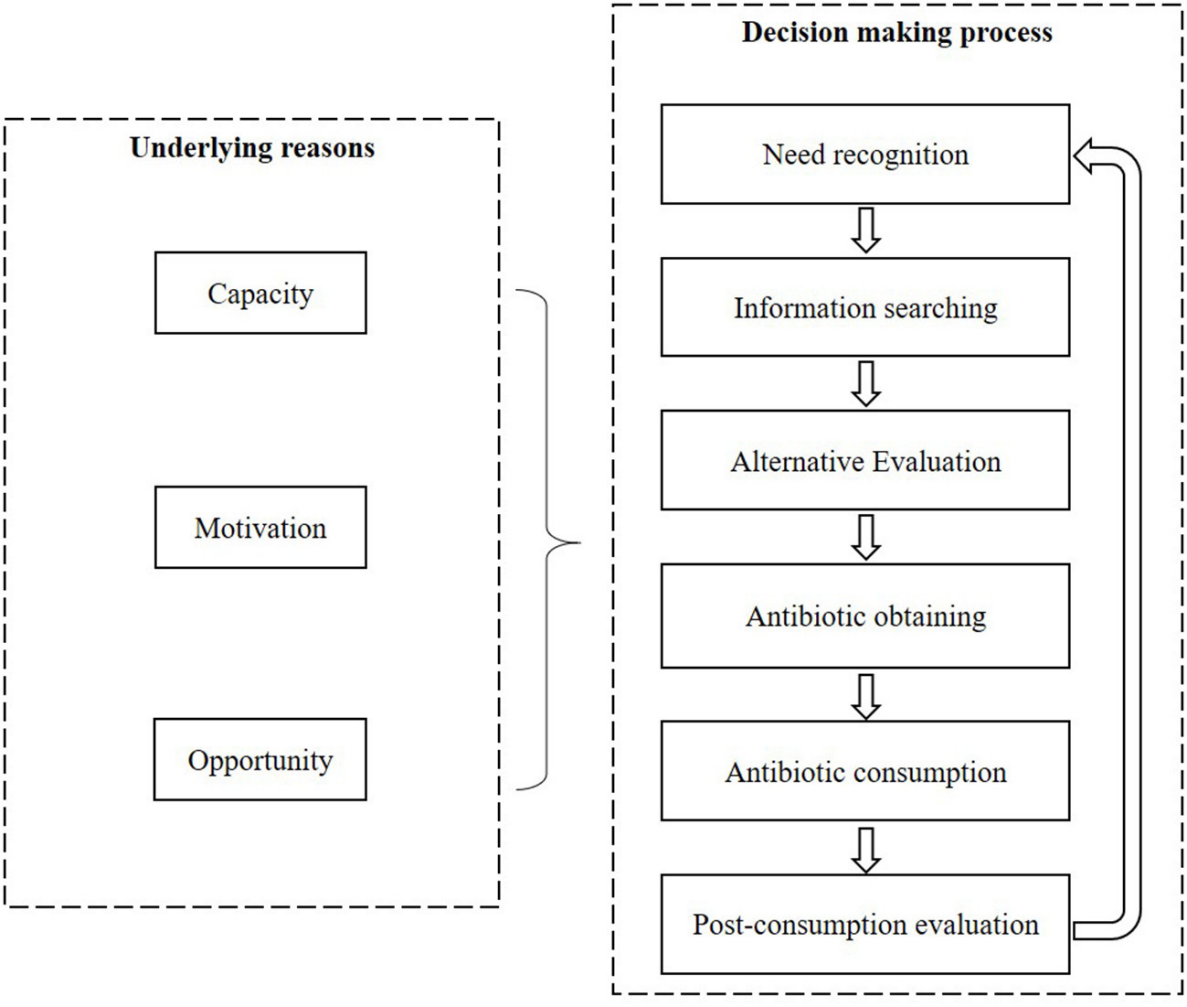
Review framework.

A mixed methods systematic review was conducted with a convergent segregated approach following the Joanna Briggs Institute (JBI) guidelines (29). The decision-making process of the public regarding antibiotic use for URTIs was described through a synthesis of the qualitative studies. The prevalence and predictors of the irrational use of antibiotics for URTIs were identified through a synthesis of the quantitative studies. The two syntheses were further integrated to generate a comprehensive understanding of the public's behaviors toward antibiotic use for URTIs. A theoretical framework was developed to guide the syntheses, incorporating the Consumer Behavior Model (CBM) (30) and the Capacity-Opportunity-Motivation Behavior (COM-B) model (31) (Figure 1).

This systematic review was pre-registered in PROSPERO (No. CRD42021266407). It forms part of a large research project in China, the protocol of which has been published elsewhere (32).

### Search strategy

Relevant publications were searched from PubMed, Cochrane Library, Embase and Web of Science from inception to 28 October 2021. The search strategy considered the Participant, Intervention, Comparison, Outcomes (PICOs) principle, using a combination of key terms associated with “user/public,” “respiratory infection,” “antibiotic/antimicrobial,” and “behavior.” Details of the search syntax were included in the Supplementary File S1.

An additional manual search was conducted through the references cited by the identified studies.

### Screening of eligible studies

Eligible studies were those peer-reviewed original research studies published in the English language. Studies that (1) were commentary and reviews, (2) were written in a language other than English, (3) did not involve antibiotic use, (4) focused only on healthcare providers, and (5) did not specify URTIs were excluded. A full list of the inclusion/exclusion criteria is presented in the Supplementary File S2.

Two researchers (CL and LD) screened the titles and abstracts of the retrieved studies against the inclusion and exclusion criteria independently. Full texts of those deemed relevant (including those disputed by the two reviewers) were extracted and further scrutinized by the same two reviewers (CL and LD) independently. Any discrepancy was resolved through discussions involving a third researcher (DW).

### Risk of bias assessment

The risk of bias of the included studies was assessed using the JBI checklist for quantitative studies (33), the Critical Appraisals Skills Programme (CASP) checklist for qualitative studies (34), and the Mixed Methods Appraisal Tool (MMAT) (35) for mixed methods studies, respectively. Two researchers (CL and LD) rated the risk of the bias score of each study independently, categorizing the studies into high-risk (0–3 for quantitative studies; 0–3 for qualitative studies; 0–5 for mixed-method studies), moderate-risk (4–6 for quantitative studies; 4–8 for qualitative studies; 6–12 for mixed-method studies), and low-risk (7–8 for quantitative studies; 9–10 for qualitative studies; 13–17 for mixed-method studies) groups. Consensus in the categorization was reached through discussions with a third researcher (DW) when any discrepancy emerged. Only the studies with a low-risk or moderate-risk of bias were included in the final data syntheses.

### Data synthesis

Data on the eligible studies were extracted into an Excel spreadsheet, covering the characteristics of study participants, methodological design and the main findings of the studies. Two reviewers (CL and LD) mapped and coded the original data into the pre-established theoretical framework (Figure 1). Any discrepancy was resolved through discussions with a third reviewer (DW) before presenting a unified version of data synthesis in line with the Preferred Reporting Items for Systematic Reviews (36).

A theory-driven thematic analysis was performed on the qualitative studies and the qualitative components of the mixed method studies. The decision-making process of the public regarding antibiotic use for URTIs was classified into six steps according to the CBM:

- Need recognition: perceptions of the nature and seriousness of URTIs;
- Information searching: information acquirement about URTI management;
- Alternative evaluation: choice of different options in URTI management;
- Antibiotic obtaining: how to obtain antibiotics;
- Antibiotic consumption: how to use antibiotics;
- Post-consumption evaluation: perceived outcomes of antibiotic use.

The data were coded under each step through an induction approach following Braun and Clarke's guidelines (37), which included data familiarization, initial coding, theme identification, and theme review.

The prevalence range of irrational behaviors of the public in antibiotic usage was reported without meta-analyses due to the high heterogeneity of the included quantitative studies (including the quantitative components of the mixed method studies). The driving forces of the irrational behaviors were categorized into three categories according to the COM-B framework (31): Capacity (individual capacity to engage in the behaviors, such as knowledge and skills); Opportunity (external factors that make individual behaviors possible, for example the availability of antibiotics); and Motivation (intrinsic drivers, such as belief and intention). The effectiveness of each category of driving forces was summarized without performing meta-analyses due to the variability in the measurements of the predictors.

### Patient and public involvement statement

Patients and the public were not involved in this systematic literature review.

## Results

### Characteristics of included studies

**Figure 2:**
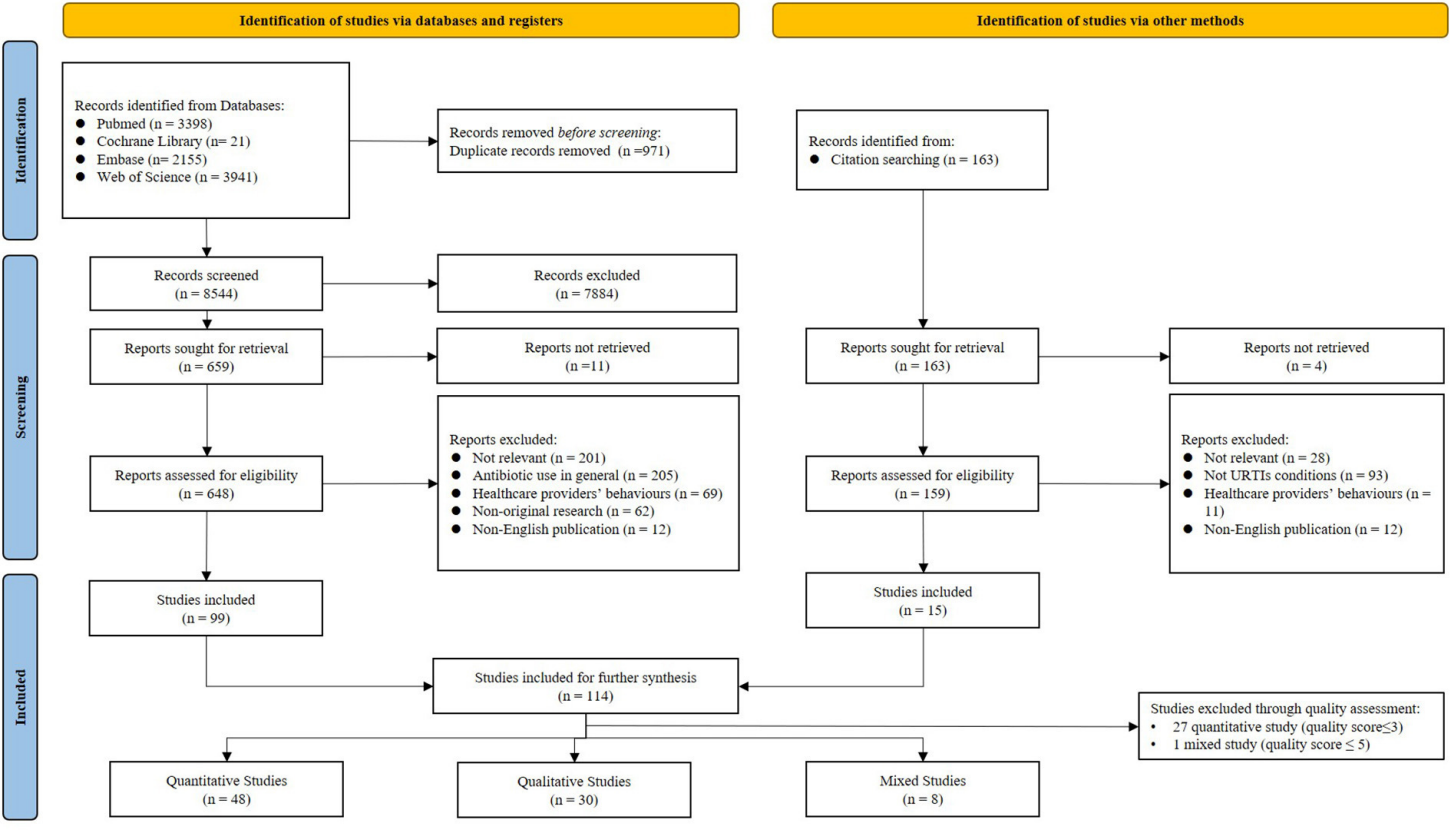
PRISMA flow diagram.

A total of 8,544 articles were identified and 114 met the inclusion criteria. The further appraisal of the risk of bias excluded 28 studies (27 quantitative and one mixed), which resulted in a final sample size of 86 studies for data syntheses: 48 quantitative, 30 qualitative, and 8 mixed methods (Figure 2).

Roughly half (*n* = 37; 43.0%) of the studies in the final sample were published after 2010 (Table 1). They were conducted in Europe (*n* = 29), Asia (*n* = 27) and North America (*n* = 21) and assessed the behaviors of patients (*n* = 46), their parents or caregivers (*n* = 31), or both (*n* = 9). Details of the characteristics of the included studies are presented in the Supplementary File S3.

**Table 1:** Characteristic of the included studies.

**Characteristic**	**Number of studies**	**Studies**
**Year of study**	
Before 2000	15	(26, 38–51)
2001–2005	11	(9, 28, 52–60)
2006–2010	13	(61–73)
2011–2015	13	(74–86)
After 2015	34	(22, 87–119)
**Study design**	
Quantitative	48	(22, 42–46, 49, 52–59, 64–72, 78–81, 96–114, 118)
Qualitative	30	(9, 26, 28, 38–41, 48, 50, 51, 60–62, 73, 74, 82–84, 86–90, 92, 94, 95, 115–117, 119)
Mix-method	8	(47, 63, 75–77, 85, 91, 93)
Region^*^	
Asia	27	(38, 46, 47, 50, 51, 54, 57, 67, 71, 72, 74, 80, 85, 96–98, 100, 101, 104–107, 110, 111, 115, 118, 119)
Europe	29	(9, 22, 26, 40, 41, 48, 52, 59, 60, 64–66, 68, 69, 73, 75, 78, 79, 81–83, 88, 89, 94, 95, 99, 108, 113, 117)
North America	21	(28, 42–45, 49, 53, 55–58, 62, 70, 84, 86, 87, 93, 102, 103, 114, 116)
Africa	6	(57, 61, 77, 90, 109, 112)
South America	2	(39, 63)
Oceanica	3	(76, 91, 92)
**Participants**	
General public	46	(9, 22, 28, 39, 40, 42, 44, 45, 49, 52, 62, 64, 66–70, 72, 74–81, 85, 87, 88, 90, 93, 95, 96, 98–100, 102, 104, 107–109, 111, 113, 115, 117, 119)
Parents or caregivers	31	(26, 38, 41, 46–48, 50, 51, 53, 55, 58, 60, 61, 63, 65, 71, 73, 82–84, 89, 97, 101, 103, 105, 106, 110, 112, 114, 116, 118)
Both general public and parents or caregivers	9	(43, 54, 56, 57, 59, 86, 91, 92, 94)
**Quality assessment**	
**Qualitative**	
Moderate	17	(28, 38–41, 50, 51, 62, 73, 74, 84, 86, 87, 90, 92, 94, 119)
High	13	(9, 26, 48, 60, 61, 82, 83, 88, 89, 95, 115–117)
**Quantitative**	
Moderate	37	(22, 42, 45, 46, 49, 52, 53, 56–59, 64, 67–72, 78–81, 98–101, 104–110, 112–114, 118)
High	11	(43, 44, 54, 55, 65, 66, 96, 97, 102, 103, 111)
**Mixed method**	
Moderate	5	(47, 63, 76, 77, 93)
High	3	(75, 85, 91)

* One study included participants from nine countries in Asia, Africa and North America.

Of the 38 studies containing a qualitative component, the majority documented need recognition (*n* = 33) (9, 26, 28, 38–41, 47, 48, 51, 60–63, 73–76, 82–91, 115–117, 119, 120), information searching (*n* = 32) (9, 26, 28, 39–41, 47, 48, 51, 60–63, 73, 74, 76, 82–84, 86–94, 115, 117, 119, 120), and an evaluation of alternative options (*n* = 35) (9, 26, 28, 38–41, 47, 48, 51, 60–63, 73, 74, 76, 77, 82–88, 90–95, 115–117, 120), while fewer studies explored antibiotic obtaining (*n* = 23) (26, 28, 38, 47, 61, 63, 74, 76, 77, 82–84, 86–88, 90, 91, 93, 95, 115–117, 120), antibiotic consumption (*n* = 15) (9, 40, 47, 50, 74, 77, 83, 85, 86, 88, 90, 93, 116, 117, 119), and post-consumption evaluation (*n* = 21) (9, 26, 47, 50, 60, 61, 74, 77, 83, 84, 86, 88–92, 94, 116, 117, 119) (Quotes in relation to the themes are presented in the Supplementary File S4).

Of the 53 studies containing a quantitative component, 18 studies (22, 42, 52–54, 63–67, 75, 78, 96–101) analyzed the care-seeking behavior of URTI patients (Table 2), 24 studies (22, 43, 44, 52, 55, 56, 58, 64, 68, 75, 91, 93, 96–99, 102–108, 114) reported public expectations/demand for antibiotics for URTIs (Table 3), 19 studies (58, 67, 69–72, 75, 78, 96–98, 100, 101, 106, 108–111, 114) documented antibiotic self-medication (including leftover antibiotics and those purchased without a prescription) for URTIs (Table 4), eight studies (57–59, 67, 75, 98, 106, 114) described the non-adherence of the public to antibiotic treatments (Table 5), and 10 studies (45, 46, 67, 79, 80, 106, 112–114, 118) surveyed the public's overall irrational use of antibiotics (Supplementary File S5). Another six studies reported other behaviors relating to the public's antibiotic use, including antibiotic storage (58, 106, 114), antibiotic treatment delay (81), and future antibiotic expectations after the use of prescribed antibiotics (52, 64) (Supplementary File S5).

**Table 2:** The public's care-seeking behaviors for URTIs and its influencing factors.

**Study**	**Behaviors**		**Influencing factors**			
	**Measured outcomes**	**Prevalence**	**Capability**	**Opportunity**	**Motivation**	**Individual characteristic**
Roope et al. (22)	Adults' care-seeking to HCPs for 5 day influenza-like-illnesses (simulated)	47.9%				
Luque et al (63)	Caregivers' care-seeking to HCPs for different clusters of URTIs symptoms	67.0–83.5%		-Having bicycle (↓) -Non-dirt floor (↑) -Having TV (↑) -Higher income (↑)		
Chai et al. (100)	Adults' care-seeking to HCPs in 12 months for RTIs	59.3%	-Limited antibiotic-related knowledge (↑) -Higher education (↓)	-Having health insurance (↓)		-Male (↑)
Andre et al. (65)	Parents' care-seeking to HCPs for 18-month-old child's RTIs in the last 1 month	22.9%			-Higher concerns of URTIs (↑)	
Carling et al. (66)	Adults' care-seeking to HCPs for a sore throat (simulated)	22.7%	-Increased knowledge of causes and treatment of sore throat (↓)		-Perceived less importance of sore throat (↓)	
Lin et al. (96)	Undergraduate students' care-seeking for URTIs in 1 month	25.1%	-Higher awareness of AMR (↓) -Medical background (↓)	-Having stored antibiotics (↑) -Having purchased antibiotics without prescription (↑)	-Perceived higher severity of URTIs (↑) -Perceived antibiotic efficacy (↑) -Presence of fever (↑) -Experienced multiple symptoms (↑)	-Living regions (Δ)
Lin et al. (97)	Parents' care-seeking to HCPs for child's URTIs in 1 month	68.6%	-Parental medical background (↓)	-Having stored antibiotics (↓) -Information sources (Δ)	-Perceived higher severity of URTIs (↑) -Perceived antibiotic efficacy (↑) -Presence of fever (↑)	
Cheng et al. (101)	Parents' care-seeking to HCPs for children's latest URTI symptoms in 1 year	78.0%		-Having stored antibiotics (↓)	-Presence with specific symptoms (↑)	
McNulty et al. (75)	Adults' care-seeking to HCPs for URTIs in 6 months	19.7%			-Perceived higher severity of URTIs (↑)	-Occupation (Δ)
Emslie and Bond (52)	Adults' care-seeking to HCPs for the most URTIs in 5 months	23.0%			- Different symptoms of URTIs (Δ) -Perceived higher severity of URTIs (↑)	- Elderly (↑)
Mainous et al. (42)	Adults' care-seeking to HCPs for 5-day sore throat, cough, and runny nose with clear discharge (simulated)	42.0%	-Higher education (↓)	-Higher family income (↓) -Health insurance (Δ)		-Race (Δ) -Elderly (↑) -Smoker (↑)
	Adults' care-seeking to HCPs for 5-day sore throat, cough, and runny nose with discolored discharge (simulated)	72.0%		-Health insurance (Δ)		-Male (↓) -Elderly (↑) -Smoker (↑)
	Adults' care-seeking to HCPs for real sore throat, cough, and runny nose with clear discharge in 1 year	31.0%				
	Adults' care-seeking to HCPs for real sore throat, cough, and runny nose with discolored discharge in 1 year	35.0%				
You et al. (67)	Adults' care-seeking to HCPs for the most recent URTIs	92.5%				
Freidoony et al. (98)	Adults' care-seeking to HCPs for URTIs in 6 months	59.3%		-Health insurance (Δ)	-Perceived antibiotic efficacy (↑) -Perceived Higher severity of URTIs (↑)	
Friedman et al. (53)	Parents care-seeking for URTIs	N/A	-Higher knowledge of URTIs and antibiotic use (↓)	-Parents' income (only for URTIs with green runny nose) (↑)		
Ivanovska et al. (78)	Adults' care-seeking for URTIs in 6 months	32.9%				
	Parents' cares-seeking for under-5 year children's URTIs in 6 months	94.5%				
Tang et al. (54)	Adults' early care-seeking to HCPs for URTIs (within 2 days after the onset of URTIs)	46.9%		-Experience of URTIs (↓) -Social class (Δ) -Without work (↑)	-Presence of fever (↑)	-Elderly (↓)
	Caregivers' early care-seeking to HCPs for children's URTIs (within 2 days after the onset of URTIs)	45.1%			-Presence of fever (↑) -Perceived higher severity of URTIs (↑) -Belief of one must consult doctor for common cold (↑)	
Osborne and Sinclair (64)	Patients' care-seeking for the most RTI in 5 months	14.1%				
Roope et al. (99)	Adults' change of care-seeking for simulated influenza-like-illness	29.1–46.1% (less/much less likely to visit HCPs) 10.3–14.1% (more/much more likely to visit HCPs)		-Provided information regarding AMR (Δ) -Surprised by provided information regarding AMR (↑)	-Perceived antibiotic efficacy (↑)	
	Parents' change of care-seeking for simulated children's influenza-like-illness	N/A		-Provided information regarding AMR (Δ) -Surprised by provided information regarding AMR (↑)	-Perceived antibiotic efficacy (↑)	

↑, Significant positive association; ↓, Significant negative association; Δ, Significant; N/A, Not available; Blank cells, No significant factor was identified.

**Table 3:** The public's antibiotic expectation behaviors for URTIs and its influencing factors.

**Study**	**Behaviors**		**Influencing factors**			
	**Measured outcomes**	**Prevalence**	**Capability**	**Opportunity**	**Motivation**	**Individual characteristic**
Braun and Fowles (43)	Adults' antibiotic expectation for cold symptoms	49.6%		-Thinking too many people use antibiotics to treat cold (↓)	-Perceived higher severity of URTIs (↑) -Belief URTIs have lasted too long (↑) -Perceived prescription medicine efficacy (↑) -Confidence about how to treat cold (↑)	
	Parents' antibiotic expectation for children's cold symptoms	30.1%			-Perceived higher severity of URTIs (↑) -Belief that wanting relief (↑) -Perceived antibiotic efficacy (↑)	
Roope et al. (22)	Adults' antibiotic demanding for 5 day influenza-like-illnesses (simulated)	38.9%		-Surprised by provided information regarding AMR (↑)	-Perceived antibiotic efficacy (↑)	-A person as very low discounter of future (↓)
Broniatowski et al. (102)	Adults' antibiotic expectation for the last URTIs	N/A			-Perceived antibiotic efficacy (↑) -Attitudes toward antibiotics that there is some chance that it could be effective (↑)	
Lin et al. (96)	Undergraduate students' antibiotic demanding for URTIs in 1 month	17.30%				
Lin et al. (97)	Parents' antibiotic demanding for child's URTIs in 1 month	7.70%	-Parents higher ability to identify antibiotics (↑)	-Having stored antibiotics (↑)	- Perceived higher severity of URTIs (↑) -Perceived antibiotic efficacy (↑) - Presence of fever (↑)	
McNulty et al. (75)	Adults' antibiotic expectation for URTIs in 6 months	53.10%				
Goggin et al. (103)	Parents' antibiotic expectation for children's ARTIs	28.30%	-Parents' limited antibiotic knowledge (↑) -Parents' improved knowledge of antibiotic use (↓) -Limited education (↑)			-Parental background (Non-English speaking) (↑) -Younger parents (↑)
Emslie et al. (52)	Adults' antibiotic expectation for different URTIs symptoms	0.3–67.2%			- Different symptoms of URTIs (Δ)	
Gaarslev et al. (91)	Adults' antibiotic expectation for a cold/flu (simulated)	19.50%	-Limited knowledge of antibiotic (↑) -Limited knowledge of AMR (↑)			- Personal background (Non-English speaking) (↑) -Younger age (↑)
	Adults' antibiotic demanding for a cold/flu (simulated)	16.90%	-Limited knowledge of antibiotic (↑) -Limited knowledge of AMR (↑) -Higher education (↑)			-Personal background (Non-English speaking) (↑) -Younger age (↓)
Faber et al. (68)	Adults' antibiotic expectation for common cold	10.50%	-Limited knowledge of AMR (↑) -Limited knowledge of antibiotics (↑) -Higher education (↓)	-Past use experience (↑)	-Perceived antibiotic efficacy (↑) -Presence of URTIs symptoms (cough, cold, sore throat and fever (↑)	
	Adults' antibiotic expectation for influenza	46.90%				
	Adults' antibiotic expectation for pneumonia	92.70%				
Davis et al. (93)	Adults' antibiotic expectation for cough/common cold	22%	-Limited knowledge of antibiotics (↑)			-Younger age (↑)
Kong et al. (104)	Elders' antibiotic expectation for different URTIs symptoms	27.9–55.7%	-Limited knowledge of antibiotics (↑)		-Perceived antibiotic efficacy (only for antibiotic expectation for cold/flu/cough) (↑)	
Saleh Faidah et al. (105)	Parents' antibiotic expectation for children's URTIs symptoms	53%			- Different symptoms of URTIs (Δ)	
El Khoury et al. (106)	Parents' antibiotic expectation for children's URTIs symptoms	15.70%				
Hernández-Díaz et al. (114)	Caregivers' antibiotic expectation for children's URTIs symptoms	14.40%				
Freidoony et al. (98)	Adults' antibiotic expectation for URTIs in 6 months	14.20%				
Mangione-Smith et al. (55)	Parents' antibiotic expectation for children's URTIs symptoms	70%			- Presence of ear pain (↑) -Higher level concerns of URTIs (↑)	-Parental background (Non-Hispanic white) (↑)
Parimi et al. (120)	Caregivers' antibiotic demanding for children's URTIs symptoms	22.60%	-Limited knowledge (↑)			
Hong et al. (44)	Patients' antibiotic expectation for URTIs symptoms	50.00%		-Past experience with URTIs and antibiotic use (↑)		
Kaplan et al. (107)	Antibiotic demanding for URTIs during visit	59.70%	-Less than college/university education (↑)	-HCPs characteristics (Δ)		-Adults patients (ref: children) (↑)
	Antibiotic demanding for URTIs during visit after an intervention for HCPs and patients	60.20%	-Less than secondary education (ref: college/university) (↑)	-Health facilities with interventions for physicians and education for patients (↑)		
Chlabicz et al. (108)	Patients' antibiotic demanding for URTIs symptoms	16.70%				
	Patients' demanding for non-antibiotic treatment for URTIs symptoms	5.50%			-Presence of different symptoms (Δ)	
Belongia. et al. (56)	Adults' antibiotic demanding for URTIs in 6 months	27.80%				
	Parents' antibiotic demanding for children's URTIs in 6 months	15.20%				
Osborne and Sinclair (64)	Patients' antibiotic expectation for different URTIs symptoms	0.4–66.5% (based on symptoms)				
Roope et al. (99)	Adults' change of antibiotic expectation for simulated influenza-like-illness	42.3–54.7% (less/much less likely to expect antibiotics) 7.5–10.1% (more/much more likely to expect antibiotics)		-Provided information regarding AMR (Δ) -Surprised by provided information regarding AMR (↑)	-Perceived antibiotic efficacy (↑)	
	Parents' change of antibiotic expectation for simulated influenza-like-illness			-Provided information regarding AMR (Δ) -Surprised by provided information regarding AMR (↑)	-Perceived antibiotic efficacy (↑)	

↑, Significant positive association; ↓, Significant negative association; Δ, Significant; N/A, Not available; Blank cells, No significant factor was identified.

**Table 4:** The public's antibiotic self-medication behaviors for URTIs and its influencing factors.

**Study**	**Behaviors**		**Influencing factors**			
	**Measured outcomes**	**Prevalence**	**Capability**	**Opportunity**	**Motivation**	**Individual characteristic**
Chai et al. (100)	Adults' non-prescription antibiotic purchasing for RTIs in 12 months	15.92%				
	Adults' lefover antibiotic use for RTIs in 12 months	13.10%				
Lin et al. (96)	Undergraduate students' antibiotic self-medication for URTIs in 1 month	16.30%		- Having stored antibiotics (↑) -Having purchased antibiotics without prescription (↑)	-Perceived higher severity of URTIs (↑) -Perceived antibiotic efficacy (↑) -Experienced multiple symptoms (↑)	-Living regions (Δ)
	Undergraduate students' antibiotic self-medication from non-prescription purchasing for URTIs in 1 month	12.50%				
	Undergraduate students' antibiotic self-medication from leftover/relatives for URTIs in 1 month	10.20%				
Lin et al. (97)	Parents' antibiotic self-medication for child's URTIs in 1 month	18.60%	-Parents higher ability to identify antibiotics (↑)	-Having stored antibiotics (↑) -Information sources (Δ)	-Perceived higher severity of URTIs (↑) -Presence of fever (↑)	
Cheng et al. (101)	Parents' antibiotic self-medication for children's latest URTI symptoms in 1 year	20.50%		-Having stored antibiotics (↑)	-Present with clear nasal discharge (↑)	-Parents higher age (↑) -Large family size (↑)
McNulty et al. (75)	Adults' leftover antibiotic use for URTIs in 6 months	0.40%				
El Khoury et al. (106)	Parents' antibiotic self-medication from non-prescription purchasing for children's URTIs	5.20%				
	Parents' antibiotic self-medication for children's fever	6.50%				
You et al. (67)	Adults' antibiotic self-medication from non-prescription purchasing for the most recent URTIs	7.30%				
	Adults' antibiotic self-medication from family/friends for the most recent URTIs	1.10%				
Hernández-Díaz et al. (114)	Parents' antibiotic self-medication from non-prescription purchasing for children's URTIs	0.00%				
	Parents' antibiotic self-medication for children's fever	6.20%				
Freidoony et al. (98)	Adults' lefover antibiotic use for URTIs in 6 months	3.50%				
Ngu et al. (109)	Adults' self-medication before care-seeking for RTIs	41.90%				-Past experience of TB (↓)
Grigoryan et al. (69)	Adults' leftover antibiotic use for URTIs in 12 months	N/A		-Living regions (Northern/Western Southern/Eastern Europe) (Δ) -Previous use of prescribed antibiotics for URTIs (↑)		
Parimi et al. (120)	Caregivers' antibiotic self-medication for children's URTIs in 30 days	33.10%			-Perceived higher severity of URTIs (↑)	
	Caregivers' antibiotic self-medication from non-prescription purchasing for children's URTIs	28.00%			- Presence of different symptoms (Δ)	
Landers et al. (70)	Adults' and parents' antibiotic self-medication for oneself or children's URTIs	23.60%	-Higher ability to identify non-antibiotic drugs for URTIs (↓)			
Togoobaatar et al. (71)	Caregivers antibiotic self-medication for children's URTIs in 6 months	42.30%	-Mothers higher knowledge of antibiotics (↓)	-Having stored antibiotics (↑) -Mothers' self-medication with antibiotics (↑)	-Tendency for antibiotic demanding (↑)	-Children's increased age (↑)
Ivanovska et al. (78)	Adults' antibiotic self-medication for URTIs in 6 months	17.80%				
	Adults' antibiotic self-medication from non-prescription purchasing for URTIs in 6 months	6.00%				
	Adults' antibiotic self-medication from leftover/relatives for URTIs in 6 months	11.80%				
	Parents' antibiotic self-medication from non-prescription purchasing for under-5 year children's URTIs in 6 months	0.00%				
	Parents' antibiotic self-medication from leftover/relatives for under-5 year children's URTIs in 6 months	1.80%				
Tan et al. (72)	Adults' antibiotic self-medication before care-seeking for URTIs	4.90%				
Chlabicz et al. (108)	Patients' antibiotic self-medication before care-seeking for URTIs	7.60%				
Hussain et al. (110)	Parents' antibiotic self-medication for children's fever	15.70%				
Luo et al. (111)	Adults' antibiotic self-medication for cough in 12 months	12.20%	-Medium knowledge of antibiotic (ref. low) (↑)	-Past experience with cough (↓)		-Age (Δ) -Having chronic diseases (↓)

↑, Significant positive association; ↓, Significant negative association; Δ, Significant; N/A, Not available; Blank cells, No significant factor was identified.

**Table 5:** The public's antibiotic adherence behaviors for URTIs and its influencing factors.

**Study**	**Behaviors**		**Influencing factors**			
	**Measured outcomes**	**Prevalence**	**Capability**	**Opportunity**	**Motivation**	**Individual characteristic**
Perez-Gorricho et al. (59)	Adults' adherence of prescribed antibiotics for RTIs	86% (daily-dosage adherence) 85% (treatment duration adherence)		-Antibiotic administration (Δ)		
McNulty et al. (75)	Adults' adherence of prescribed antibiotics for URTIs in 6 months	74.3%				
El Khoury et al. (106)	Parents' adherence of antibiotic treatment for children's URTIs	80.4% (daily-dosage adherence) 89.2% (treatment duration adherence)				
You et al. (67)	Adults' adherence of antibiotic treatment for the most recent URTIs	78.5%				
Hernández-Díaz et al. (114)	Caregivers' adherence of antibiotic treatment for children's URTIs	72.2% (daily-dosage adherence) 83.5% (treatment duration adherence)	-Higher education (↑)			
Freidoony et al. (98)	Adults' adherence of antibiotic treatment for URTIs in 6 months	64.3%				
Parimi et al. (120)	Caregivers' adherence of antibiotic treatment for children's URTIs	67.7%				
Pechere (57)	Adults' and caregivers of children's adherence of antibiotic treatment for RTIs	69.0% (53–90% based on countries)				

↑, Significant positive association; ↓, Significant negative association; Δ, Significant; N/A, Not available; Blank cells, No significant factor was identified.

### Decision-making process of the public regarding antibiotic use for URTIs

**Figure 3:**
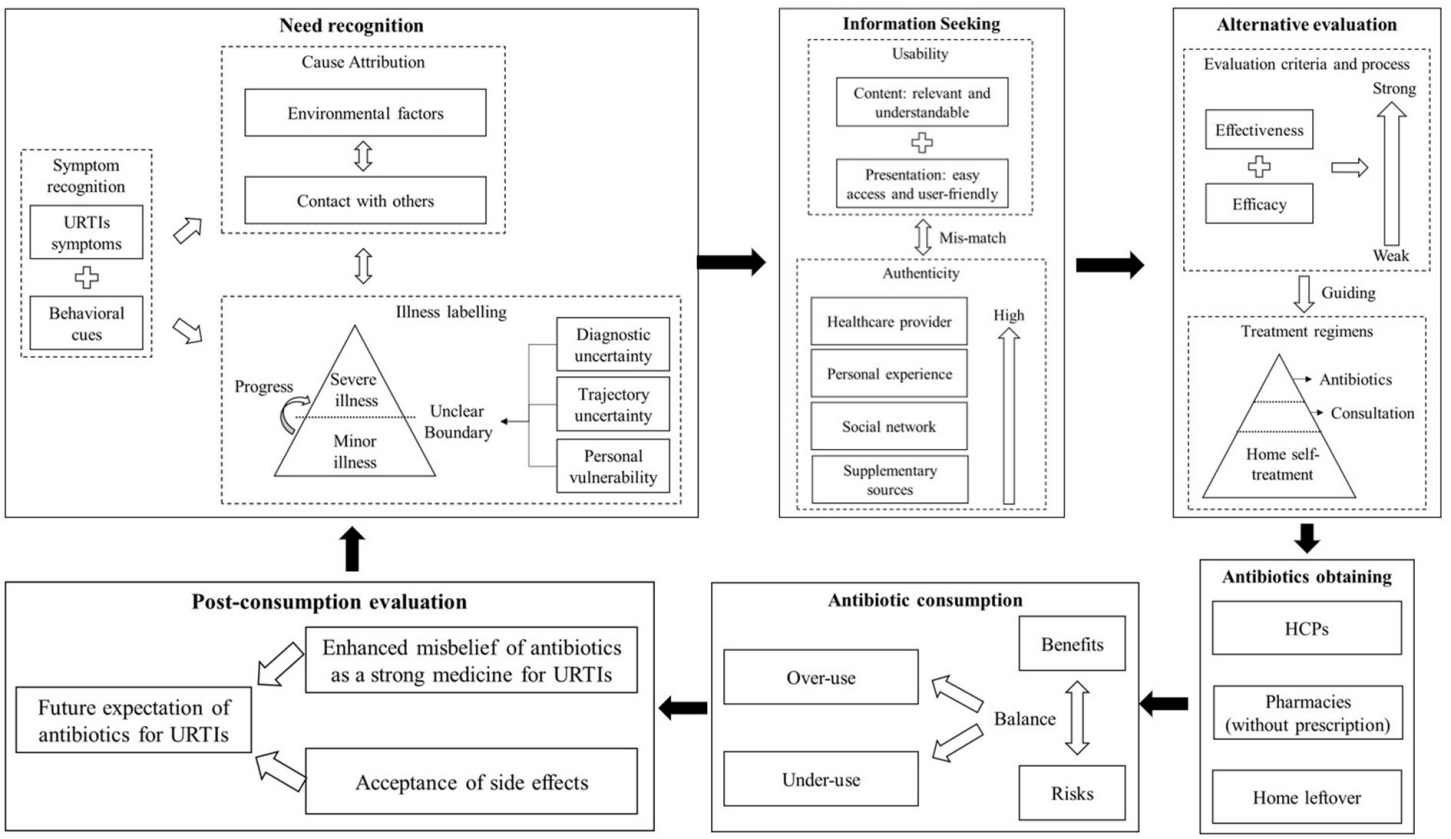
Qualitative synthesis of the public's antibiotic use for URTIs based on CBM.

Eleven themes emerged covering the six CBM stages: need recognition, information searching, alternative evaluation, antibiotic obtaining, antibiotic consumption, and post-consumption evaluation. The six stages reinforce each other, resulting in a vicious cycle (Figure 3).

### Stage one: Need recognition

This is the first stage in which people self-assess the need for taking further actions. It involves symptom recognition, cause attribution, and illness labeling.

#### Symptom recognition

URTIs are highly recognizable, as patients usually experience typical symptoms (26, 38, 39, 50, 73), such as cough, nasal discharge, sore throat and fever. The public also see declines in energy levels (fatigue) and appetite as an indication of URTIs (41, 50, 62, 73, 82). These “presence” or “absence” symptoms are not mutually exclusive and often co-contribute to the self-assessment of the patients (47, 48, 50, 51, 82).

#### Cause attribution

URTIs are considered infectious. Environmental factors (38–40, 47, 50, 60, 76, 88, 90, 115) and close contacts (38, 40, 50, 51, 60, 88, 90, 115) are perceived as major causes of URTIs. Exposure to cold weather, wind and rain without proper protection is linked to increased risk of URTIs (38–40, 76, 90, 115). URTI patients are deemed infectious, but not necessarily linked to the virus (38, 47, 51, 60, 76, 115). The two causes are not seen as mutually exclusive, but each one can result in URTIs with or without the other (39, 60, 76).

#### Illness labeling

Labeling is a critical step that shapes decisions for further actions. URTIs can be labeled as a minor or severe condition (9, 39, 40, 50, 51, 73, 76, 84) depending on the combination and seriousness of the “presence” and “absence” symptoms (41, 47, 50, 51, 82). There is no clear boundary between minor and severe, which makes it difficult for people to assign their own condition to one of the binary categories (9, 26, 50, 73, 82–84). Although URTIs are commonly considered to be a minor condition (40, 60, 62, 75, 76, 86, 90), there is a belief that it can progress into a severe case if not treated properly (39, 41, 51, 87, 115).

Concerns about diagnostic uncertainty and trajectory uncertainty contribute to the labeling (73). The former represents a concern about missing clues relating to the seriousness of the condition, while the latter represents a concern about the potential long-term impact of the condition (73). The occurrence of an alarming symptom (such as rash, convulsion, chesty cough or fever) (38, 40, 41, 48, 50, 51, 60, 63, 73, 74, 82, 84, 85, 88, 91, 116, 117, 119), a lack of response to treatments, and worsening symptoms (38, 41, 48, 50, 51, 62, 63, 73, 82, 86) are common clues for the labeling of severe conditions. A longer than anticipated duration of symptoms (41, 50, 73, 75, 82, 84, 86–88, 90, 91, 116, 117, 119) and functional impairment of daily lives (41, 75, 88, 90, 119) may attract serious concerns about the trajectory of URTIs.

The quantitative studies confirmed that the perceived seriousness of symptoms (52, 54, 66, 96, 98), the presence of certain specific symptoms (52, 54, 96, 97, 101) or multiple symptoms (97), disruptions to daily lives (75), and strong concerns about the impact of URTI (65) are major predictors of patients seeking medical attention for URTIs (Table 2).

Individual vulnerability is also taken into consideration in the labeling. A lower threshold of severe labeling is adopted for vulnerable populations such as children (40, 50, 73, 82, 83, 88, 90).

### Stage 2: Information seeking

Information acquisition can shape or change people's understanding and attitudes toward URTIs and its treatment. Two major themes were identified in association with the impacts of acquired information: authenticity and usability.

#### Authenticity

People often feel that it is difficult to obtain helpful information for decision-making regarding URTIs (82). Multiple sources of information (62, 82) come from healthcare providers (9, 26, 40, 47, 48, 73, 76, 82, 83, 86, 88, 90, 91, 93, 94, 115, 117, 119), patients (9, 28, 41, 47, 48, 50, 60–63, 73, 74, 82–84, 86–88, 90, 91, 94, 115, 117, 119), individual social networks (9, 26, 39, 47, 51, 62, 63, 73, 82, 87, 90, 91, 93, 94, 117), and the public media (26, 73, 74, 82, 87–90, 93, 94, 117). These are not always consistent and can be confusing.

The public usually gives the highest authenticity to information provided by healthcare providers (HCPs) (26, 93, 94), in particular in relation to options for different treatments for URTIs (9, 26, 76, 82, 93). HCPs are also the final word in resolving discrepancies in information obtained from various sources (82, 86). However, poor communication between HCPs and patients is common (26, 48, 82, 86, 88, 91–93, 115). HCPs tend to offer minimal interpretation of key messages, such as the nature (virus infection/self-limiting illness) of the diagnosis (26, 91) and the appropriate use of prescribed medicines (90, 91), leaving room for patient misinterpretation (26, 40, 76, 90). For example, if a patient is complaining of a rattling sound in their chest when breathing, but the physician declares that their chest is clear, this does not ease the concerns of the patient, rather it generates further confusion and anxiety (26, 91). Adding to the complexity are the differences, sometimes contradictions, in the management of URTIs by HCPs, even with the same HCP over time. This jeopardizes people's ability to make sense of the actions of the HCP and derive a clear knowledge for future use (26, 91, 93, 119).

Lessons learnt from personal experience serve as another important source of information (9, 28, 41, 47, 48, 50, 60–63, 73, 74, 82–84, 86–88, 90, 91, 94, 115, 117, 119). These lessons are highly valued by the public and can shape their views on the effectiveness (or a lack of) of different treatments for URTIs (9, 28, 48, 62, 74, 82, 83, 86, 117). As a result, the authority of HCPs can sometimes be challenged by patients despite their limited ability to make a scientific interpretation of their personal experiences (28, 86). Indeed, personal experiences may result in a misbelief in certain treatments simply due to the self-limiting nature of UTRIs (9, 117). Quantitative studies have established associations between past experiences of antibiotic use with higher expectations of antibiotic prescriptions (44, 68), self-medication with antibiotics (69, 71), and a combination of irrational antibiotic use behaviors (106, 113, 114) (Tables 3, 4; Supplementary File S5).

People also seek information from others through their social networks (9, 26, 39, 47, 51, 62, 63, 73, 82, 87, 90, 91, 93, 94, 117). This can be selective. The credibility of the information is judged based on the background of the person being consulted. Information from those who have a health background (26, 47, 62, 73, 82, 90, 94), have accumulated extensive experience in managing URTIs (82, 94), and who are deemed to have a close relationship with the person seeking the information (38, 94) is more likely to be given higher authenticity. However, antibiotic resistance and the rational use of antibiotics are scantly discussed on social network platforms (94).

People can get access to relevant information via traditional [e.g., books (26, 82)] or/and modern media channels [e.g., the Internet (82, 90, 93, 117) and social media (26, 74, 88, 89, 94, 117)]. However, information from these sources is likely to be contradictory and generate suspicions (82, 89). People may decide to only use information found on social media for managing minor conditions to minimize potential risks (89).

#### Usability

Useful information needs to have valid content (26, 48, 51, 82, 88–90, 93) and be easy to access (26, 82, 89). The URTI-related information searched for by the public includes how to differentiate between a severe and minor condition, what to do and what not to do, and how to prevent future occurrence (26, 48, 82). The content has to be presented in a way which is easy to understand (51, 88, 90), balancing basic technical jargon and lay language (26). The communication channels need to be user-friendly (26) and free of financial and technological barriers in relation to accessibility (26, 82, 89).

### Stage 3: Alternative evaluation

At this stage, people use their knowledge and gathered information to evaluate alternative approaches to URTI management.

#### Evaluation criteria and process

People are aware of the existence of a diverse range of treatment regimens for URTIs including traditional therapies, over-the-counter (OTC) medicines, and antibiotics (38, 40, 41, 47, 48, 50, 51, 60, 62, 63, 73, 74, 76, 77, 82–88, 90, 93, 94, 115–117). Antibiotics are not considered to be a distinct medicine in comparison with other allopathic medicines (26, 47, 61, 74, 76, 121). Instead, they are assessed along with other drugs and are ranked on a continuum ranging from weak to strong (38, 40, 41, 48, 50, 73, 84, 85, 87, 88, 115), depending on their effectiveness and efficacy (61, 74, 77, 85, 88, 91, 117) in relieving symptoms (61). Weaker treatment regimens for minor conditions and stronger treatment regimens for severe conditions are deemed appropriate (26, 38–41, 47, 48, 50, 51, 61, 85, 117).

#### Treatment regimens

The treatment regimens adopted by patients with URTIs can be grouped into three categories, namely home remedies, HCP-prescribed treatments, and antibiotics.

Home remedies are considered appropriate for minor conditions (38–41, 50, 51, 63, 73, 84, 87, 88, 90, 115), but not for severe conditions (63). These can involve a variety of medicines, both traditional/natural and OTC allopathic (63, 88, 90). Home remedies are commonly accompanied by continuous monitoring of changes in symptoms to inform the need for upgrading action (48, 63, 73).

Consultations with HCPs are anticipated when the condition is labeled as severe or the symptoms are worsening despite the application of a home remedy (40, 82, 86, 87, 90, 94, 95, 116). The purpose is to obtain a clear diagnosis (including URTIs and other diseases) (40, 82, 86, 116) and the correct treatment (82, 90, 116). Sixteen quantitative studies show that the proportion of people seeking medical consultations for URTIs varies considerably, ranging from 14.1 to 94.5%. Those who are not trained medical professionals (96, 97), have attained limited education (42, 100), have limited knowledge of antibiotics and URTIs (53, 66, 96, 100), have purchased antibiotics without a prescription in the past (96), perceive a severe condition (52, 75, 96, 97), and hold a misbelief in relation to antibiotic efficacy (96–99) are more likely to seek a consultation with HCPs than others, while other tested predictors generated mixed results (Table 2).

Antibiotics are perceived as a strong treatment regimen for severe URTIs (26, 41, 50, 74, 76, 77, 84, 85, 88, 89, 91, 93, 95, 116, 117). Such a view is consistent across different populations (77, 116). Perceived severity is a significant predictor of care-seeking (52, 75, 96, 97) (Table 2), expectations of antibiotic prescriptions (43, 55, 97) (Table 3), and self-medication with antibiotics (58, 96, 97) (Table 4) according to the quantitative studies. The side effects of antibiotics are considered to be common but relatively benign (47, 50, 77, 86, 116). Some people believe that antibiotics can shorten the duration and halt the progress of URTIs (74, 76, 77, 83, 88, 91, 92, 95), and thus, warrant use even for minor conditions. Three quantitative studies (72, 108, 109) revealed that 4.9–41.9% of adult patients adopt self-medication with antibiotics before seeking formal care from HCPs (Table 4).

It is evident that most people do not understand how antibiotics function (76, 77, 91, 93, 95, 117) and cannot distinguish between different kinds of antibiotics (60, 116). The strength of antibiotics is usually rated higher based on their stronger odor, higher price, novelty, and parenteral route of administration (50, 77). Although some people know that antibiotic products only kill bacteria (84, 117), misunderstandings about the etiology of URTIs can still lead to a false expectation of antibiotics (40, 76). Patients can also attach added value to antibiotic treatment which goes beyond the management of their current condition, such as reducing the uncertainty of health events to come (74, 76, 77, 83, 88, 91), a reward for patient's efforts (91), and a shortcut to returning to normal life (86, 91). Furthermore, HCPs who prescribe antibiotics are viewed as caring for patients (26, 60, 76, 84, 91). Perceived added value has broadened the scope of antibiotic use.

### Antibiotics obtaining

Antibiotics can be obtained from prescriptions or pharmacy retail outlets (without a prescription), or they can be left over from previous prescriptions.

Although not all patients expect HCPs to prescribe antibiotics for URTIs (38, 41, 48, 61, 63, 75, 82, 83, 88, 90, 91, 117), conscious and unconscious pressures are exerted on HCPs (28, 86). Apart from direct requests, some patients suggest antibiotics as one of the options, depict a dire picture of the severity of their symptoms, highlight daily life implications (e.g., a prolonged journey), and refer to the past success of antibiotic treatments (28). The percentage of URTI patients who expect antibiotic prescriptions ranges from 0.3 to 92.7% (Table 3), as reported in 24 quantitative studies. Eight studies (22, 56, 58, 91, 96, 97, 107, 108) revealed that between 5.5 and 60.2% of URTI patients requested antibiotic prescriptions directly. Two studies (43, 56) showed that parents are less likely to demand antibiotic treatment for URTIs for their children (15.2–30.1%) compared with adult patients with URTIs (27.8–49.6%).

Perceived effectiveness and added value are the major reasons for requesting antibiotics, according to qualitative studies (28, 76, 82, 86, 88, 91, 115, 116). Such a demand can also be justified by the patient as their consumer right (91, 115). The quantitative studies show that misbelief in antibiotic efficacy (22, 43, 68, 97, 99, 102, 104) is a reliable predictor of high expectations of antibiotics (Table 3). Furthermore, those who perceive they have a severe condition (43, 97) [the presence of certain specific symptoms (52, 55, 68, 97, 105, 108) and prolonged duration of symptoms (43)] are more concerned about their illness and have a higher expectation of antibiotics (55). In addition, the increased awareness of AR (68, 91), a higher knowledge level of antibiotics (68, 91, 93, 103, 104) and higher education attainment (68, 91, 107) are also associated with lower antibiotic expectations.

Overall, the public feels it is increasingly difficult to obtain antibiotic prescriptions for URTIs (26, 84, 88). However, they mainly blame cost-saving pressures on HCPs for these difficulties (26, 83, 91). When HCPs fail to prescribe antibiotics as expected by the patients, most patients choose to suppress their demands (76, 87, 88, 116). Some may request an explanation (93). A few may insist on being prescribed antibiotics (84, 91), possibly through a doctor shopping strategy (76, 88, 115).

In some systems, antibiotics can be obtained from pharmacy retail outlets without a prescription (38, 47, 50, 61, 74, 76, 77, 88, 115, 117). People do so for a variety of reasons, but not necessarily because of prescribing barriers. Perceived antibiotic efficacy (88, 117), limited value of HCP consultations (117), easy access (38, 47, 74), and poor knowledge of the prescribing policy (74) are the underlying reasons for purchasing antibiotics at a pharmacy without a prescription. The antibiotic products purchased are usually chosen by the patients themselves (61, 117) based on their personal experience (50, 95, 117) and advice from their family, colleagues and community pharmacists (76, 77, 117), depending on the price and availability of the products (47, 61). Four quantitative studies (67, 78, 96, 100) reported that between 6.0 and 15.9% of URTI patients self-medicated using purchased antibiotics without a prescription (Table 4). Two studies reported that between 5.2 and 28.0% of parents purchased antibiotics without a prescription to treat their children's URTI (58, 106), while two other studies reported that no parents self-medicated their children's URTIs with antibiotics in the US (114) and the Republic of Macedonia (78).

Leftover antibiotics are very likely to be used for similar conditions, including URTIs (61, 87, 88). Three studies show that between 6.7 and 24.5% of parents or caregivers store antibiotics at home (58, 106, 114) (Supplementary File S5). Another six quantitative studies show that between 0.4 and 13.1% of URTI patients self-medicate with leftover antibiotics (67, 75, 78, 96, 98, 100) (Table 4).

In terms of the overall prevalence of antibiotic self-medication, 11 studies show that the rate ranges from 6.2 to 42.3% (Table 4). Mongolia has the highest rate (42.3%) (71) followed by those from Trinidad and Tobago (58) (33.1%), the US (70, 114) (6.2–23.6%), China (96, 97, 101, 111) (12.2–20.5%), the Republic of Macedonia (78) (17.8%), Saudi Arabia (110) (15.7%), and Lebanon (106) (6.5%).

Perceived severity [intensity of symptoms (58, 96), presence of a specific symptom (58, 97, 101) or multiple symptoms (96, 97)], and a misbelief in antibiotic efficacy (96, 97) are major predictors of self-medication with antibiotics for URTIs, which is facilitated by easy access to antibiotics (71, 96, 97, 101).

### Antibiotic consumption

URTI patients take a pragmatic approach and adjust the uptake of antibiotics through self-assessment of their conditions. They weigh the perceived benefits against risks continuously (9, 83, 88) based on their own interpretation such as changes in symptoms (see stage 1: need recognition) (9, 47, 50, 74, 88, 117). They may increase the dosage or extend the duration of antibiotic treatment in the case of a lack of anticipated improvement (9, 74, 117). Once the symptoms disappear (47, 50, 88), they may terminate antibiotic use earlier than prescribed due to concerns about potential side effects (47, 77, 85) or simply because of an unfavorable attitude toward medications in general (47, 119). However, the criterion of what time frame is “too long” to be taking medication varies (50).

Five quantitative studies (57, 58, 67, 75, 98) reported an overall patient adherence to antibiotic instructions of between 67.3 and 78.5% (Table 5). Another three studies (59, 106, 114) differentiated between daily dose adherence (ranging from 72.2 to 86%) and treatment duration adherence (ranging from 83.5 to 89.2%). Low levels of education (114) and complex antibiotic administration (59) are associated with low patient adherence. Some studies show that poor adherence to instructions on antibiotic use is associated with an unawareness of its importance (93), mis-interpretation of the HCPs (47, 50, 90), and financial burdens (47, 50). Increased awareness of the side effects of antibiotics does not necessarily reduce adherence but increases vigilance (86).

### Post-consumption evaluation

Personal experience of antibiotic use for URTIs feeds into future decision making. Recovering from URTIs whilst taking antibiotics can enhance the misbelief that antibiotics are a strong medicine for URTIs and can lead to an acceptance of the side effects of antibiotics.

#### Enhanced misbelief of antibiotics as a strong medicine for URTIs

URTIs are a self-limiting condition. Relief of symptoms is considered an indication of the effectiveness of medicines, including antibiotics (61). This enhances the misbelief that antibiotics are a strong medicine for URTIs (9, 117). Such a misbelief can be exaggerated if antibiotic prescribing for URTIs is highly prevalent (9, 117). The high prescribing of antibiotics in HCPs can be a justification for the misbelief about antibiotics and normalizes the practice (9). This raises expectations on the future use of antibiotics for URTIs (28, 50, 83, 86, 117), resulting in a vicious cycle (9). Two studies (52, 64) show that between 74.40 and 81.80% of those who have been prescribed with antibiotics for URTIs expect antibiotic treatment for URTIs in the future (Supplementary File S5).

#### Acceptance of side effects

People are aware of the potential side effects of antibiotics, including concerns about antibiotic resistance. Such knowledge can be obtained from their personal experience (61, 74) or through other sources (89, 91, 116). However, it is usually a vague understanding (9, 61, 74, 88–91, 116). Some may believe antibiotic overuse is harmless (47, 60, 74, 89), failing to link it to antibiotic resistance (74, 89). Of those who admit the association between antibiotic overuse and antibiotic resistance (60, 89), most believe it is an individual matter of low response to antibiotics (26, 60, 61, 74, 77, 84, 89–92, 94, 116, 119), rather than bacteria developing antibiotic resistance beyond individuals (74, 89, 92, 94, 116). They may consider themselves at a low risk of antibiotic resistance simply because they are low users (89, 91, 92, 116).

There is optimism for the future. Many believe that new antibiotics will be available to deal with antibiotic resistance (74, 89, 91, 92), or the human body will learn to adapt to this situation (89). Instead of worrying about the future, priority is given to the recovery of the current conditions (91). Although low antibiotic resistance if preferrable (60, 94), people do not know how to address this issue (89, 91) since they tend to see themselves as low users and blame others for the problem (116). Awareness of antibiotic resistance is unlikely to deter antibiotic use for URTIs (60, 88, 91).

People are also aware of the other side effects of antibiotics (86, 91, 92), such as skin rashes and diarrhea. However, these are deemed benign (86). Some may even see these side effects as an indication of strong medicine (61). The experience of side effects is unlikely to deter people from the future use of antibiotics for URTIs (91).

## Discussion

### Main findings

This mixed methods systematic review identifies several types of presentations in the public's irrational use of antibiotics for URTIs, including antibiotic expectations, demand for antibiotic prescriptions, self-medication with antibiotics, purchase of antibiotics without a prescription, and non-adherence to medical instructions. The synthesis of the quantitative data indicates widespread irrational antibiotic use for URTIs internationally and highlights the importance of the motivation factor as a driving force. High levels of concerns about the severity of the condition and misbelief in the efficacy of antibiotics for treating URTIs were consistently found to be associated with the irrational use of antibiotics, whereas, individual capacity and opportunity, the other two factors corresponding to the COM-B framework, showed mixed effects in the existing studies.

The synthesis of the qualitative data generated eleven themes in line with the six CBM stages: need recognition, information searching, alternatives evaluation, antibiotic obtaining, antibiotics consumptionand post-consumption evaluation. The public are confident in recognizing URTIs. They tend to categeorize their condition into a minor or severe case without using a clear set of criteria. They obtain information from a wide range of sources and those derived from their own personal experiences and from the HCPs are the most valued in their decision making. Potential treatments are rated based on perceived effectiveness and efficacy and antibiotics are highly likely to be rated as a strong medicine for URTIs. The public also attach additional value to antibiotics beyond treatment for URTI. Apart from prescriptions obtained from HCPs, some patients also purchase antibiotics from pharmacy retail outlets without a prescription and use leftover products for self-medication. The continuation or suspension of antibiotic uptake usually depends on self-assessment of the benefits against the risks, leading to poor adherence to medical instructions (if applicable). Because of the self-limiting nature of URTIs, the public are likely to misattribute their recovery to the effect of antibiotic treatments, reinforcing their belief that antibiotics are a strong medicine for URTIs. Although they are often aware of the association between antibiotic use and AR, they tend to perceive AR as being of little relevance to themselves, especially when they consider themselves to be a low user of antibiotics. There is optimism in the public in relation to human responses to AR, which downplays individual responsibilities. These actions form a vicious cycle, making it a very challenging task to reduce antibiotic use for URTIs.

### Strengths and limitations

To the best of our knowledge, this is the first mixed methods systematic review on the public's use of antibiotics for URTIs. The synthesis of data was guided by the COM-B (for quantitative data) and CBM (for qualitative data) frameworks. Further integration of the quantitative and qualitative findings has helped us to comprehensively understand the public's behaviors in relation to antibiotic use for URTIs. The public's behaviors over the entire process, which starts from symptom recognition and ends with a final reflection of personal experience, form a vicious cycle, fueling public expectations on antibiotic use for URTIs. The findings have significant implications for policy development and interventional designs.

This review focuses on antibiotic use for URTIs due to its high prevalence and the importance of tackling the irrational use of antibiotics. Attempts to generalize the results to other conditions should be cautious. In this study, we did not perform meta-analysis on the quantitative studies because of the high heterogeneity of the findings. We also note that there is a lack of standardized approaches in monitoring the public use of antibiotics, leading to varied measurements.

### Interpretation

This review shows that the public overuse of antibiotics for URTIs is prevalent worldwide. This result is consistent with the findings of the public's antibiotic use in general (12, 122). However, URTIs are a self-limiting condition usually caused by virus, for which antibiotic treatment is not recommended (123). It is common for people to treat URTIs using antibiotics with or without a prescription.

The underlying reasons for the public overuse of antibiotics are multifaceted according to the COM-B framework. There is consistent evidence to support the association between individual motivation and the overuse of antibiotics. However, no definite conclusions can be made for the roles of individual capacity and opportunity: the results of the existing studies are mixed. It is likely that their effects are context-dependent. In most settings, high levels of individual capacity (education, medical background, knowledge of antibiotic use and AR) have been found to be associated with the appropriate use of antibiotics (58, 68, 70, 71, 79, 91, 93, 103, 104, 106, 107, 111, 113, 114). However, the public understanding of the value of antibiotics can be vague and sometimes wrong (76, 77, 91, 93, 95, 117), which can result in the irrational use of antibiotics (45, 80, 97, 111).

The recent series of studies in the UK (22, 99) shows that the effect of improved individual capacity on the rational use of antibiotics is moderated by the motivation factor. With a misbelief in antibiotic efficacy for URTIs, a high awareness of AR may even result in increased care-seeking and demand for antibiotic treatments for URTIs (99). Misbelief in antibiotic efficacy is common in settings where antibiotics are often prescribed for treating URTIs (9, 26, 76, 82, 93) and patients have become used to such treatments (9, 28, 48, 62, 74, 82, 83, 86, 117). Easy access to antibiotic products is linked to high expectations of antibiotics (44, 68, 97) and high prevalence of self-medication with antibiotics (69, 71, 96, 97, 101). In contrast, in settings where there are high concerns of the overuse of antibiotics (43) and where people are well-informed by verified reliable sources (97, 107), a positive link between individual capacity and the rational use of antibiotics is evident.

This review decomposes the decision-making process of the public regarding antibiotic use for URTIs into six intertwined stages in line with the CBM framework. They reinforce each other, forming a vicious cycle. The inappropriate use of antibiotics is facilitated by the misinterpretation of the illness condition fueled by misinformation obtained from multiple sources (including past experience of antibiotic use for URTIs) and easy access to antibiotic products. Health regulators, HCPs, consumers, and social media all play a role.

In countries where antibiotics have been frequently used for treating URTIs, consumers tend to exert great pressures on HCPs to prescribe antibiotics (43, 44, 55, 105, 107). Quite often, there also exist loopholes in the sales of prescription-only medicines in these countries, that is consumers may be able to purchase antibiotics from pharmaceutical retail outlets without a prescription (8). Adding to the complexity are the perverse financial incentives that encourage HCPs to prescribe antibiotics for URTIs (124). Because URTIs are a self-limiting condition, consumers may misattribute their recovery to antibiotic use (9, 117), further enhancing their expectations of antibiotics for treating URTIs (52, 64). This expectation is also justified by an over-optimistic view on novel antibiotic development (74, 89, 91, 92) and a lack of perceived individual responsibility in curbing antibiotic resistance (89, 91, 92, 116). Modern information technology has made access to and the sharing of information easy. But unfortunately, it also increases the dissemination of misinformation (82, 89), which not only drives consumer demand for antibiotics, it also erodes their trust in HCPs (28, 86). High levels of antibiotic consumption are often accompanied by high expectations, leading to leftover or non-prescription purchasing antibiotic products to be used for self-medication (69, 71, 96, 97).

HCPs are highly valued by the public for their medical advice, including advice in relation to URTIs (26, 93, 94). It is undeniable that irrational prescribing has played an important role in driving the overuse of antibiotics. However, both restrictive (125) (e.g., prescribing restrictions) and persuasive (126, 127) (e.g., training, public reporting, peer support) interventions targeting HCPs often generate limited effects without involving measures targeting consumers. Public interventions often take a traditional education approach, lacking innovation and evidence of its effectiveness (128).

### Policy and practice implications

The findings of this study have significant policy and practice implications. A systems approach is needed to break the vicious cycle in the public overuse of antibiotics for URTIs. Interventional measures must consider the interactions between HCPs and consumers and address the issues relating to all the six steps in consumer decision making.

The public should be educated to take responsibility to protect the health and wellbeing of not only themselves but also society through the rational use of antibiotics (81). Social marketing campaigns need to be innovative and specific, empowering consumers to make an appropriate assessment of their illness conditions and adequate choice of care, targeting various stages of decision making (129). High public awareness of the serious consequences of AR and its association with the individual use of antibiotics is urgently needed (130). In some countries, this implies a dramatic shift in consumer culture (8). It is important to note that AR is also associated with inappropriate antibiotic use in agriculture and on animals (131).

Intervention strategies targeting misinformation are critical to enable consumers to make proper decisions regarding antibiotic use (132). HCPs are well-positioned in fighting misinformation as they enjoy the highest authority in medical matters. However, their role goes beyond patient education. Antibiotic prescribing practices of HCPs can directly shape the views, experience, and expectations of patients in relation to antibiotics (26, 91, 93, 119). There are a growing number of Internet-based channels run by government agencies and HCPs to provide evidence-based information for consumers (132). Consumer uptake of these sources of information depends on the level of public trust (81).

Regulations and law enforcement need to be strengthened in some countries (8). Many antibiotic products are low priced, which has helped improve the accessibility of essential medicines. However, they should never be sold in pharmacy retail outlets without a prescription.

## Conclusion

The overuse of antibiotics in the public for URTIs is prevalent worldwide. It results from both an over-prescription from HCPs and self-medication by consumers. Consumers are motivated to use antibiotics by a series of intertwined factors that form a vicious cycle. These include misinterpretation of the illness condition, misinformation shared by others, over-prescription by HCPs, poor adherence to medical advice, easy access to antibiotic products, and misjudgment of personal experience. A systems approach is required to break the vicious cycle. HCPs share the responsibility in re-shaping public attitudes toward antibiotic use for URTIs. Their communication and prescribing practices form an integral part, but not all of the decision process of the public. This calls for interventions concerning both supply-side and demand-side drivers.

## Data availability statement

The original contributions presented in the study are included in the article/Supplementary material, further inquiries can be directed to the corresponding author.

## Author contributions

CheL: conceptualization, data curation, formal analysis, funding acquisition, investigation, methodology, resources, software, supervision, validation, visualization, writing—original draft, and writing—review and editing. ChaL: conceptualization, investigation, supervision, and writing—review and editing. XZ: conceptualization, investigation, resources, supervision, and writing—review and editing. LD: formal analysis, data curation, methodology, project administration, software, validation visualization, and writing—original draft. DW: formal analysis, data curation, methodology, and project administration. RL and PQ: data curation, formal analysis, and project administration. All authors have read and agreed to the published version of the manuscript.

## Funding

This study was funded by the National Natural Science Foundation of China (grant no. 71904053). The funding body played no part in the study design, collection, analysis and interpretation of data, writing of the manuscript or the decision to submit the manuscript for publication.

## Conflict of interest

The authors declare that the research was conducted in the absence of any commercial or financial relationships that could be construed as a potential conflict of interest.

## Publisher's note

All claims expressed in this article are solely those of the authors and do not necessarily represent those of their affiliated organizations, or those of the publisher, the editors and the reviewers. Any product that may be evaluated in this article, or claim that may be made by its manufacturer, is not guaranteed or endorsed by the publisher.

## Abbreviations

AR: Antibiotic Resistance
CASP: Critical Appraisals Skills Programme
CBM: Consumer Behavior Model
COM-B: Capacity-Opportunity-Motivation Behavior
HCPs: Healthcare Providers
JBI: Joanna Briggs Institute
MMAT: Mixed Methods Appraisal Tool
OTC: Over-The-Counter
PICOs: Participant, Intervention, Comparison, Outcomes
URTIs: Upper Respiratory Tract Infections.
