# Supplementary material for: The vicious cycle of the public's irrational use of antibiotics for upper respiratory tract infections: A mixed methods systematic review
Source: Front Public Health. 2022 Oct 20;10:985188. doi: 10.3389/fpubh.2022.985188 (PMC9632431; doi:10.3389/fpubh.2022.985188)
Supplement: Supplementary file 1 [file Data_Sheet_1.PDF]

Supplementary file S1 - Search strategies

Database	Search Strategy
**Pubmed**	(("outpatient*"[Title/Abstract] OR "the public"[Title/Abstract] OR "people*"[Title/Abstract] OR "population*"[Title/Abstract] OR "consumer*"[Title/Abstract] OR "customer*"[Title/Abstract] OR "user*"[Title/Abstract] OR "purchaser*"[Title/Abstract] OR "citizen*"[Title/Abstract] OR "resident*"[Title/Abstract] OR "inhabitant*"[Title/Abstract] OR ("outpatients"[MeSH Terms] OR ("population"[MeSH Terms] OR "population groups"[MeSH Terms]) OR "population groups"[MeSH Terms]))<br>AND<br>(("knowledge*"[Title/Abstract] OR "aware*"[Title/Abstract] OR "understand*"[Title/Abstract] OR "attitude*"[Title/Abstract] OR "view*"[Title/Abstract] OR "percept*"[Title/Abstract] OR "perceiv*"[Title/Abstract] OR "opinion*"[Title/Abstract] OR "belie*"[Title/Abstract] OR "concern*"[Title/Abstract] OR "fear*"[Title/Abstract] OR "accept*"[Title/Abstract] OR "perspect*"[Title/Abstract] OR "worr*"[Title/Abstract] OR "concep*"[Title/Abstract] OR "determina*"[Title/Abstract] OR "stimulus*"[Title/Abstract] OR "incent*"[Title/Abstract] OR "reason*"[Title/Abstract] OR "motiv*"[Title/Abstract] OR "rationa*"[Title/Abstract] OR ("health knowledge, attitudes, practice"[MeSH Terms] OR "patient medication knowledge"[MeSH Terms] OR "awareness"[MeSH Terms] OR "comprehension"[MeSH Terms] OR "attitude"[MeSH Terms] OR "attitude to health"[MeSH Terms] OR "culture"[MeSH Terms] OR "health belief model"[MeSH Terms] OR "fear"[MeSH Terms] OR "concept formation"[MeSH Terms] OR "generalization, stimulus"[MeSH Terms] OR "motivation"[MeSH Terms]))<br>AND<br>("anti bacterial agents"[MeSH Terms] OR "anti infective agents"[MeSH Terms] OR ("antibiotic*"[Title/Abstract] OR "antimicro*"[Title/Abstract] OR "antibact*"[Title/Abstract] OR "anti infect*"[Title/Abstract]))<br>AND<br>("respiratory tract infection*"[Title/Abstract] OR "respiratory infection*"[Title/Abstract] OR "upper respiratory infection*"[Title/Abstract] OR "upper respiratory tract infection*"[Title/Abstract] OR ("cold*"[Title/Abstract] OR "rhinitis*"[Title/Abstract] OR "cough*"[Title/Abstract] OR "sore throat*"[Title/Abstract] OR "pharyngitis*"[Title/Abstract] OR "flu"[Title/Abstract] OR "influenza*"[Title/Abstract]) OR ("runny nose*"[Title/Abstract] OR "nasal congestion"[Title/Abstract] OR "sneez*"[Title/Abstract]) OR ("respiratory tract infections"[MeSH Terms] OR "pharyngitis"[MeSH Terms] OR "common cold"[MeSH Terms] OR "rhinitis"[MeSH Terms] OR "influenza, human"[MeSH Terms] OR "rhinorrhea"[MeSH Terms] OR "sneezing"[MeSH Terms] OR "cough"[MeSH Terms])))) AND (english[Filter])

**Web of Science**	TI=(knowledge* OR aware* OR understand* OR attitude* OR view* OR perception* OR perceiv* OR opinion* OR belie* OR concern* OR fear* OR accept* OR perspect* OR worr* OR concept* OR determina* OR stimulus* OR incent* OR reason* OR motiv* OR rationa*) OR AB=(knowledge* OR aware* OR understand* OR attitude* OR view* OR perception* OR perceiv* OR opinion* OR belie* OR concern* OR fear* OR accept* OR perspect* OR worr* OR concept* OR determina* OR stimulus* OR incent* OR reason* OR motiv* OR rationa*)<br>AND<br>TI=(Antibiotic* OR antimicro* OR antibact* OR antiinfec*) OR AB=(Antibiotic* OR antimicro* OR antibact* OR antiinfec*)<br>AND<br>TI=(outpatient* OR "the public" OR people* OR population* OR consumer* OR customer* OR user* OR purchaser* OR citizen* OR resident* OR inhabitant*) OR AB=(outpatient* OR "the public" OR people* OR population* OR consumer* OR customer* OR user* OR purchaser* OR citizen* OR resident* OR inhabitant*)<br>AND<br>TI=(respiratory tract infection* OR respiratory infection* OR upper respiratory tract infection* OR upper respiratory infection* OR cold* OR rhinitis* OR cough* OR sore throat* OR pharyngitis* OR flu* OR influenza* OR runny nose* OR nasal congestion OR sneez*) OR AB=(respiratory tract infection* OR respiratory infection* OR upper respiratory tract infection* OR upper respiratory infection* OR cold* OR rhinitis* OR cough* OR sore throat* OR pharyngitis* OR flu* OR influenza * OR runny nose* OR nasal congestion OR sneez*)
**Embase**	antibiotic OR antimicrobial OR antibacterial in Title Abstract Keyword AND public OR outpatient OR population OR people OR customer OR user OR resident in Title Abstract Keyword AND respiratory tract infection OR common cold OR pharyngitis OR rhinitis OR cough OR influenza OR rhinorrhea OR sneezing OR sore throat OR runny nose OR nasal congestion in Title Abstract Keyword AND knowledge OR attitude OR practice OR behavior in Title Abstract Keyword **-** (Word variations have been searched)
**Cochrane Library**	1. (antibiotic or antimicrobial or antibacteria).mp. [mp=title, abstract, heading word, drug trade name, original title, device manufacturer, drug manufacturer, device trade name, keyword heading word, floating subheading word, candidate term word]<br>2. (outpatient or people or population or consumer or customer or purchaser or citizen or resident or inhabitant or user or public).mp. [mp=title, abstract, heading word, drug trade name, original title, device manufacturer, drug manufacturer, device trade name, keyword heading word, floating subheading word, candidate term word]<br>3. (respiratory tract infection or upper respiratory infection or cold or rhinitis or cough sore throat or pharyngitis or flu or influenza or runny nose or nasal congestion or sneezing).mp. [mp=title, abstract, heading word, drug trade name, original title, device manufacturer, drug manufacturer, device trade name, keyword heading word, floating subheading word, candidate term word]<br>4. (knowledge or awareness or understanding or attitude or view or perception or perceive or opinion or belief or concern or fear or acceptance or perception or worry or concept or determinant or stimulus or incentive or reason or motives or ratinale).mp. [mp=title, abstract, heading word, drug trade name, original title, device manufacturer, drug manufacturer, device trade name, keyword heading word, floating subheading word, candidate term word]<br>5. 1 and 2 and 3 and 4

Supplementary file S2 - Inclusion and Exclusion Criteria

	Inclusion Criteria	Exclusion Criteria
**Language**	English	Other language
**Time period**	Inception of databases to 10th, May, 2021	Everything else
**Population**	General public, caregivers of children (age < 18 years old), outpatients, adults, elderly and consumers	Healthcare providers (physician, pharmacist, nurse and etc.) or other populations with medical background
**Study setting**	No restrictions	None
**Illness**	Indicated URTIs diagnosis;<br>Indicated URTIs symptoms (cough, sore throat, runny nose, nasal congestion, headache, fever and etc.);<br>Indicated RTIs with URTIs symptoms;	Non-indicated conditions (Antibiotic use in general);<br>Indicated other conditions (Malaria, diarrhea, UTI and etc);
**Outcome**	Studies covered at least one of the following aspects:<br>1) Need recognition: people's perception of URTIs and how they understand URTIs;<br>2) Information searching: how people obtain and understand information for URTIs treatments;<br>3) Alternative evaluation: how people evaluate different URTIs treatments and what are the assessment results<br>4) Antibiotic obtaining: how people obtain antibiotics<br>5) Antibiotic consumption: how people assess antibiotic during consumption and how they alter their behaviors<br>6) Post-consumption evaluation: people's positive and negative assessment of antibiotic use and how this influences their future use	1) Not involved people's antibiotic use practices;
**Study design**	Original research studies (quantitative, qualitative and mix-methods)	Editorials, commentaries, reviews or literature reviews, descriptive studies, poster abstracts
**Others**		Full-text article cannot be downloaded

Supplementary file S3 - Extraction and detailed characteristics of included studies

Reference	Regions	Countries	Participants	Study design	Aim	Participants	Data collection	Data analysis	Quality of study
Rutebemberwa, E.et al. (2009)	Africa	Uganda	Parents or caregivers	Qualitative	To explore caretakers' use of drugs, perceptions of drug efficacy and preferred providers for febrile children	• Four FGDs (each with 9-12 participants):<br>→ Fathers or mothers had children below five years and were known residents in sub-counties close to or far away from the district headquarters;<br>• Eight KIIs<br>→ Two are health workers from two of the local health facilities (one being government and another NGO); two with attendants in drug shops, two with attendants in private clinics and two with people who had been functioning as community medicine distributors before.	• Focus group discussions (FGDs)<br>→ An FGD guide focused on:<br>1) drugs that caretakers give children with fever or fever and cough, the drugs they consider efficacious, those they consider non-efficacious and why;<br>2) which providers caretakers go to when the children present with fever and why they go to such providers.<br>• Key informant interviews (KIIs)<br>→ The guiding questions for the KIIs:<br>1) identification of the drugs, which the caretakers (such as parents) used for malaria and for pneumonia, which of the drugs caretakers considered efficacious and why;<br>2) health providers to whom caretakers went to when their children were febrile and why they preferred such providers	Content and thematic analysis	10/10
Norris, P.,et al. (2011)	Oceanica	New Zealand	General population	Mixed	To explore the extent of Samoan people's knowledge and to describe their understanding of antibiotics	• For qualitative part, purposeful sampling via informal network was used (18 in Samoa and 13 in New Zealand) and the sample was biased toward those with some form of tertiary education;<br>• For quantitative part, 232 Samoan people attending health care facilities in Samoa and in New Zealand were surveyed (120 in Samoa and 112 in New Zealand). (No sampling information was detected)	• For qualitative part, participants' knowledge and use of antibiotics, in the context of their understandings of health and illness were explored. The topics includes: 1) Beliefs about and responses to illness; 2) Access to health services; and 3) Antibiotics; (See Article Appendix for details);<br>• For quantitative part, A questionnaire based on literature review and qualitative results were generated. The questionnaire consisted of 18 questions, including 6 demographic questions and others concerns people's knowledge, attitudes and use of antibiotics, (See Article Appendix for details)	• Qualitative: phenomenologica l approach to identify themes;<br>• Quantitative: Descriptive analysis based on Excel;	11/17
Braun, B.L.,et al. (2000)	North America	U.S.A	General population & Parents or caregivers	Quantitative	To characterize people who want antibiotics for cold symptoms and to suggest reasons for antibiotic expectations	• Participants: 505 people, 249 parents of children and 256 adults with cold symptoms;<br>• Sampling: Three primary care clinics identified eligible patients. Consecutive patients were enrolled at each site during 4 weeks in the spring of 1997 (March 10 to May 16).<br>• Inclusion criteria: primary complaint of cold symptoms, such as rhinitis, cough, fever, or sore throat. (Also several exclusion criteria, such as age, severity and etc. see articles for details)	• A trained interviewer conducted the telephone survey 48 to 96 hours after the patient's initial medical system contact;<br>• Outcome measures: Respondents were asked if they had wanted a prescription for antibiotics when they contacted the medical care;<br>• Other variables:<br>1) Background patient information;<br>2) patient's respiratory signs and symptoms (presence of a cough, fever, and nasal drainage);<br>3) rated the severity of the respiratory symptoms;<br>4) the number of days between the date of symptom onset and the date of medical contact;<br>5) cold-related beliefs;<br>6) reasons for contacting the medical care system;<br>7) their history of cold-related sequelae,<br>8) cold-related medical experiences, and<br>9) current nonmedical situational needs, such as employment concerns.	Chi-square analyses, t tests and stepwise logistic regression analysis	7/8

Roope, L.S.J.,et al. (2018)	Europe	U.K	General population	Quantitative	To investigate what drives patient expectations of antibiotics for ILI and particularly whether AMR awareness, risk preferences or time preferences play a role.	* A total of 2,064 adults in the United Kingdom;<br>* The sample was provided by Survey Sampling International (SSI), a data collection and market research company.<br>* They claimed the sample represents of adult members of the general public in terms of sex, age, ethnicity and geographic region (not confirmed)	* A self-designed questionnaire by discussion from experts, patients and the publics;<br>* Instrument measures whether participants would go to a doctor and request antibiotics if they have influenza-like-illnesses for 5 days;	Multi-variables regression	6/7
Ellis, J.,et al. (2019)	Europe	England	General population & Parents or caregivers	Qualitative	The aim of this study is to provide an evaluation of individuals' attitudes and behaviors towards antibiotics and the social network influences on these in the process of help seeking for self-limiting illnesses.	* 10 parents and 14 adult patients;<br>* Purposive a sampling strategy (biased to White British & lived in areas of low deprivation.);<br>* Participants (>=16) or parents of individuals aged ≤15 years have received a prescription for antibiotics in the last 3 months.	* Semi-structured interviews with personal community mapping exercise 1. First, mapping was used to identify personal social network;<br>2. Second, interviews regarding to: 1) the role and importance of network members; 2) the individual's attitudes and behaviors towards antibiotics drawing on their most recent experiences, 3) the influences of the identified network members.	Grounded theory	8/10
Medina-Perucha, L., et al.(2020)	Europe	Spain	General population	Qualitative	To explore the experiences and concerns of service users with ALRTIs, in relation to access to healthcare, antibiotic use and health education	* 29 Adults diagnosed with an ALRTI in the last six months (selective and purposive sampling):	* Semi-structured interviews:<br>The topics cover: 1) experiences of ALRTIs; 2) conceptualization of symptoms and language used; 3) red flag symptoms and reasons for healthcare consultations; 4) healthcare services attended for ALRTIs; 5) treatment experiences; 6) preferences and types of treatments;<br>7) knowledge of antibiotic resistance; 8) healthcare professionals' communication skills; 9) preferences and needs for written materials on ALRTIs.	Thematic Content Analysis	9/10
Manderson, L.,et al. (2020)	Africa	South Africa	General population	Qualitative	To explore the provision and use of antibiotic drugs as understood by adult patients, adults presenting with children, and health providers, including both general practitioners and nurse practitioners.	1. Observation of doctor-patient and nurse-patient consultation for colds and flu;<br>2. 65 semi-structured interviews of patients (biased to female)<br>3. 8 doctors, 15 nurses and 2 pharmacists interview;<br>4. 12 key informants interviews (senior clinicians and research scientists, government programme officers and pharmacists)	Observation and interviews	Thematic analysis	8/10
Van Hecke, O.,et al. (2019)	Europe	England	Parents or caregivers	Qualitative	To explore parents' perceptions and understanding of antibiotic use and resistance in the context of their young child with an acute RTI and explores the acceptability of strategies aimed at parents to reduce unnecessary antibiotic consumption.	* 23 Parents or carers of preschool children who had a recent acute RTI.<br>* Purposive sample	Semi-structured interviews:<br>Interview guideline:1) caring for a child with an RTI, 2) beliefs about antibiotics, 3) understanding of antibiotic resistance and 4) views on current public antibiotic awareness campaigns at the time; 5) parents' perceptions of attitudes of significant others (e.g. family and friends) and beliefs about confidence in carrying out specific behaviors (e.g. self-care of RTI).<br>Guidelines mainly based on Social Cognitive Theory	thematic analysis	9/10

Luque, J.S.,et al. (2008)	South America	Ecuador	Parents or caregivers	Mixed	To identify the factors determining timely maternal health-seeking for ARIs	Care givers of children under five & healthcare providers	Convenience sampling;<br>1. Focus group discussions with 60 mothers. Topics covers 1) barriers to seeking a doctor, 2) home treatments for common childhood illnesses, and 3) local illness terms during summer 2004;<br>2. In-depth interviews with 25 healthcare providers. Topics covers: standard care for children with ARI; prescribing antibiotics; delays in care-seeking for ARI; perceptions of mothers' knowledge regarding the signs and symptoms of ARI; operating hours and staff needs; patient costs; and patient volume<br>3. Quantitative data: Semi-structured questionnaire of 91 female caregivers	Thematic analysis & descriptive analysis	12/17
Jin, C.,et al. (2011)	Asia	China	General population	Qualitative	To answer 1) How do the villagers perceive antibiotics and antibiotics resistance? 2) When and how do the villagers use antibiotics? and 3) What strategies do villagers adopt in order to maximize the benefits from antibiotics and to minimize the risks that they themselves identify?	1. 28 in-depth interviews among villagers;<br>2. 12 focus group discussions (Three with village committee, four with poorer villagers, five with wealthier villagers)	Focus group discussion and in-depth interviews:<br>Topics cover: (a) drug (antibiotics) use and knowledge; (b) health seeking behavior; (c) perception of doctors in different kinds of health facilities; (d) fake drugs; and (e) any medical disputes experienced.	Thematic analysis	7/10
Broniatowski, D.A.,et al. (2018)	North America	U.S.A	General population	Quantitative	To determine whether patients' perceptions for antibiotic use influence their reported antibiotic expectation and use	1. 519 online adult participants (years over 18)<br>2. 240 emergency department patients;<br>3. 155 primary care health care providers	Self-developed questionnaire with 46 likert scale items	Factor analysis of questionnaire and regression analysis	7/7
Scott, J.G.,et al. (2001)	North America	U.S.A	General population	Qualitative	To analyze the effects of physician-patient communication on unnecessary antibiotic prescribing based on direct observation of outpatient visits to family physicians for ART infections.	298 patients with ART infections	Field researchers directly observed and dictated descriptions of approximately patients encounters with clinicians.	Induction by an expert group	7/10
Perez-Gorricho, B.,et al. (2003)	Europe	Europe (France, Germany, Italy and Spain)	General population & Parents or caregivers	Quantitative	To determine patient attitudes and behaviors to antibiotic management of mild-to-moderate RTIs	2436 Adults or 818 parents whose child had mild-to-moderate respiratory tract infection and antibiotics had been prescribed in 2 months before survey	Questionnaire survey covers: the socio-economic status of the interviewee, the severity of the infection for which the antibiotic was prescribed and its impact on their lifestyle. Interviewees were also asked about their perceptions of their doctor, his/her general manner and management of the illness. In addition, general questions were asked of the subjects about their doctor's age and gender.	Mean comparison methods (T-test, chi-square and etc)	6/8

Chai, J.,et al. (2019)	Asia	Anhui, China	General population	Quantitative	To describe help seeking behavior from a medical doctor and antimicrobial use for common infections among rural residents of Anhui province, China.	2611 rural residents	A structured questionnaire survey covers:<br>1) the prevalence of symptoms of common infections,<br>2) help-seeking from medical doctors in any setting,<br>3) recall of antimicrobial prescription for those infections<br>4) other potential determinants (online supplementary Appendix 1).	Regression analysis	5/8
Tillekeratne, L.G.,et al. (2017)	Asia	Sri Lanka	General population	Qualitative	To assess Sri Lankan patients' and physicians' attitudes towards ARTI diagnosis and treatment	* 25 pediatric patients and 25 adult patients with influence-like disease (WHO definition);<br>* 5 outpatient department physicians;	Semi-structured interviews:<br>Patient interviews cover: 1. Care-seeking patterns for ARTIs; 2. Knowledge of ARTI etiology and treatment; 3. Assessment of treatment received at the OPD visit;<br>Physician interviews cover: 1. Approach to the diagnosis and management of ARTIs; 2. Physicians' reasons for potential antibiotic over-prescription for ARTIs; 3. Physicians' understanding of antibiotic resistance and impact of resistance on antibiotic prescribing patterns; 4. Opportunities for improving the care of patients with ARTIs in the OPD	Thematic analysis	9/10
Andre, M.,et al. (2007)	Europe	Sweden	Parents or caregivers	Quantitative	To describe families with high, medium and low concern about infectious illness with regard to social variables, perception of infection proneness, belief in antibiotics and relate the concern for infectious illness to morbidity, physician consultations and antibiotic prescriptions for the 18-month-old child in the family.	818 parents having 18 months old child (recruited from health clinics by nurses, consecutively sampling with exclusion criteria)	Questionnaire survey covers:<br>1. Demographics;<br>2. Concerns for infectious illness (perceived high/medium/low infectious illness threat)<br>3. Belief in antibiotics;<br><br>Log book:<br>Ask parents to note all their child's infectious symptoms during 1 month day by day.	Logistic regression with mean comparison	8/8
Carling, C.L.,et al. (2009)	Europe	Norway	General population	Quantitative	To assess to which the use of different graphical displays effect choices about whether to go to the doctor for antibiotics for a sore throat.	1760 participants<br>(Sampling using TV broadcast on Norway)	RCT design with simulated scenarios and using questionnaire survey for data collection (detailed design):<br>1. Participants were simulated that they had a sore throat;<br>2. They were divided into five groups with different presentation of information: 1) face icons; 2) bar graphs; 3) bar graphs with different content; 4) bar graphs with added information; 5) no information;<br>3. Participants were surveyed on 1) relative importance of the discomfort of a sore throat, 2) side effects of antibiotics, 3) recurrence of sore throat, 4) inconvenience of getting and taking antibiotics; These value measures were calculated as relative importance score (RIS, higher RIS is expected with higher likelihood of deciding to go to the doctor);<br>4. They viewed the different graphical presentation of information (pros and cons of taking antibiotics) or no information;<br>5. They then would be asked whether they would or not go to a doctor for antibiotics;<br>6. They then were asked to rate different presentations of information (preferences);	Logistic regression with mean comparison	7/7

							7. After received informed information of sore throat, they would reconsider the choice of whether go to a doctor for antibiotics;		
Helman, C.G.,et al. (1978)	Europe	England	General population	Qualitative	To explain why many folk concepts of illness remain unchanged, and to describe how biomedical treatment and concepts (for example, the germ theory of disease) are easily incorporated into the folk model without challenging its basic premises. In fact, they may serve to reinforce it	Personal experience and interviews with patients, district nurses, receptionists, and with seven of my general practitioner colleagues	No details	No details	4/10
Lin, L.,et al. (2020)	Asia	China	General population	Quantitative	To investigate the decision-making process of Chinese university students with respect to antibiotic use for upper respiratory tract infections (URTIs).	2834 Undergraduate students who had URTI symptoms<br>(using cluster random sampling method)	Questionnaire survey covers:<br>1. Knowledge about antibiotics and resistance: 1) AMR awareness (five items), 2) ability to identify antibiotics (seven items) and misconception about the antibiotics as 'anti-inflammatory drugs' (one item);<br>2. Perceived severity of URTIs: knowing that URTIs are self-limiting or not;<br>3. Perceived antibiotic efficacy: antibiotics' efficacy to treat URTIs (5 items)<br>4. Cues to action: (a) self-diagnosed severity of current infection, measured by numbers of cold symptoms experienced. (b) Presence of fever.<br>5. Access to antibiotics: (a) Keeping antibiotics at home in the past year. (b) Over-the- counter purchase of non-prescription antibiotics in the past year.<br>6. Point of care: from hospitals above county level, county level hospitals, township hospitals and private clinics.	Regression analysis	7/8

Lin, L.,et al. (2021)	Asia	China	Parents or caregivers	Quantitative	To investigate the extent and risk factors associated with the likelihood of (1) self-medication with non-prescription antibiotics by parents; (2) healthcare seeking; and (3) parental requesting for antibiotics and unnecessary prescriptions by healthcare providers in children with self-diagnosed URTI symptoms	3188 parents with children aged 0-13 years who had URTI symptoms (using a multistage cluster random sampling)	Questionnaire survey convers: 1) parental sociodemographic information; 2) healthcare and antibiotic-related knowledge and perceptions; 3) last episode of URTI symptoms experienced by the child within the past month, 4) treatment and parental care-seeking process and behaviors for the child's illness (i.e., the chemical or brand names of antibiotics obtained from clinics and retail pharmacies).	Regression analysis	7/8
Hudelson, P.,et al. (1995)	South America	Bolivia	General population	Qualitative	To describe people's 'explanatory models" for respiratory infections.	Two rural communities: 1) a Quechua-speaking community of about 180 families located in the department of Cochabamba; 2) an Aymara-speaking community of 63 families in the department of La Paz.	The FES focuses on families' and health care providers' perceptions and practices concerning symptom recognition, diagnosis, home treatment and health care seeking for ARI, and consists of a series of unstructured and structured research procedures. These include: 1. Semi-structured interviewing with key informants, 2. Narratives of past ARI episodes, 3. Clinic interviews with mothers of children who currently have ARI, 4. Several formal ethnographic techniques (free listing, paired comparisons, matching and rating tasks), 5. Hypothetical illness scenarios, 6. Inventories of medicines found in the home. 7. Interviews with health practitioners.	No details	7/10
Morgan, K.,et al. (2009)	North America	U.S.A	General population	Qualitative	To learn more about the values, beliefs, and attitudes held by rural individuals and families regarding ARIs	5 Focus group with 42 participants （8 key informant interviews were used to validate the results)	5 Focus group with 42 participants using semi-structured interview questions (purposive sampling with contactors in each community)	Thematic analysis	8/10
Kresno, S.,et al. (1994)	Asia	Indonesia	Parents or caregivers	Qualitative	To identify local beliefs, perceptions, and practices surrounding acute respiratory infections (ARI) in infants and young children.	1) 13 key informants (HCPs, mothers, community men, traditional healers), 2) 50 mothers who had children under age five years 3) 13 health care providers (various occupation including traditional healers), 4) 50 mothers seeking care for a child under five years of age who was currently sick with an acute respiratory ailment.	Various methods but seemed mainly interviews (structured and semi-structured)	No details	7/10
Deschepper, R.,et al. (2002)	Europe	Netherlands & Belgium	General population	Qualitative	To understand how different consumption levels of antibiotics are related to cross-cultural differences in lay perspective towards disease labelling, initial	11 participants in Belgium (representing 36 family members) and 13 in The Netherlands (representing 33 family members); a quota sample method was used;	A semi-structured interview, focusing on dealing with signs and symptoms of URTD and on attitudes towards initial coping with illness and the use of antibiotics After interviews, a 3-month diary for recording all URTD episodes experienced by the participant as well as all family members (if applicable), the signs and symptoms of illnesses, treatments and actions undertaken.	Thematic analysis and constant comparative method	10/10

					coping strategies and antibiotics utilization				
Cheng, J., et al. (2019)	Asia	Anhui, China	Parents or caregivers	Quantitative	To gather quantitative data on and provide an overview of the full range of acts by caregivers as they manage children through their URTI illness experiences	925 Residents caregivers (mothers or fathers) of under 15 years old children (using a stratified-cluster randomized sampling in rural villages)	A structured questionnaire survey covers:<br>(a) demographic characteristics of caregivers;<br>(b) caregivers' general knowledge and their general behavior related to antibiotics;<br>(c) the recent experience with children's URTI symptoms	regression analysis	5/8
Ingram, J., et al (2013)	Europe	U.K	Parents or caregivers	Qualitative	To explore parents' views on support and information needs prior to consulting when children have RTIs to inform an intervention to support future parental decision-making.	7 focus groups and 30 semi-structured interviews were held with 60 parents (with children aged 5 months - 17 years)<br>* 30 in focus group only;<br>* 23 in interviews only;<br>* 7 in both activities;	7 focus groups, guidelines covering parents' pre-consultation beliefs and behaviors.<br>30 interviews, guidelines covering parents' perceptions and experiences of primary care consultations when their child had a cough.	Thematic analysis and constant comparison technique	9/10
Cabral, C., et al (2015)	Europe	U.K	Parents or caregivers	Qualitative	To explore some of the drivers for parental consulting and clinician antibiotic prescribing for children with RTIs	7 focus groups and 58 semi-structured interviews were held with 60 parents (with children aged 5 months - 17 years) and 28 clinicians<br>* 30 in focus group only;<br>* 23 in interviews only;<br>* 7 in both activities;<br>* 28 clinicians in interviews	7 focus groups: guidelines covering parents' pre-consultation beliefs and behaviors.<br>30 interviews for parents: guidelines covering parents' perceptions and experiences of primary care consultations when their child had a cough.<br>28 interviews of clinicians focused on:<br>* Usual practice in managing child with acute cough<br>* Influences on prescribing decision<br>* Communication practices<br>* Clinician needs	Thematic analysis and constant comparison technique	9/10
Finkelstein, J. A., et al. (2013)	North America	U.S.A	Parents or caregivers	Qualitative	To examine current beliefs about common infections and antibiotic use among parents of diverse backgrounds and educational levels.	5 focus groups with 31 parents having 1 or more children aged 6 years or younger	Focus groups using semi-structured guide with open-ended questions (no details of the guide), based on aims, it may cover:<br>1) to explore care seeking and use of home remedies for common infections,<br>2) knowledge and attitudes regarding antibiotic use, and<br>3) issues of trust in medical providers.	Content analysis with the principles of the immersion/crystal lization method	8/10
Jónsson, H.,et al. (2002)	Europe	Iceland	Parents or caregivers	Qualitative	To deal with the feelings of parents of pre-school children with newly diagnosed acute otitis media and their views regarding the disease and its diagnosis and treatment.	23 parents of pre-school children with newly diagnosed acute otitis media (Consecutive and purposive sampling)	A semi-structured interview. Guidelines cover:<br>1. The disease process.<br>2. Parents' ideas on otitis media.<br>3. Treatment, medication and non-medication.<br>4. The family's support system.<br>5. The parents and other background information.	Grounded theory method	9/10
Wun, Y. T., et al. (2012)	Asia	Hong Kong, China	General population	Mixed	To investigate whether the attitudes and behavior of parents' use of antibiotics for themselves were	8 Focus groups with 56 participants, aged 20–73 years (purposive sampling)<br>2471 general populations aged over 18 years old (convenience sampling)	No details of the guideline of group discussions and the content of questionnaire	Thematic analysis and mean comparison methods	16/17

					different to that for their children.				
Szymczak, J. E., et al. (2018)	North America	U.S.A	Parents or caregivers	Qualitative	To better understand what parents think about the risks and benefits of antibiotics immediately before their child is seen by a pediatrician for symptoms consistent with an ARTI.	109 Parents who had a child between 3 months and 18 years of age that had at least 1 chief complaint consistent with an ARTI (eg, sore throat, ear pain, congestion, fever, cough).<br>(Purposive sampling)	A semi-structured interview guide covers two aspects:<br>1) Open-ended questions intended to get parents to share their reasons for bringing their child to the doctor, expectations for the visit, and opinions about antibiotics with minimal prompting;<br>2) Closed-ended questions designed to assess parental concern about antibiotic risks, treatment failure, and adverse effects	Thematic analysis	9/10
McNulty, C. A., et al. (2013)	Europe	England	General population	Mixed	To build up a profile of the ‘RTI clinical iceberg’ by exploring how the general public manage RTI, visit GPs and why.	1. Qualitative part:<br>17 Responders who had recently had an RTI, were opportunistically recruited in four pharmacies in England;<br>2. Quantitative part:<br>1767 adults aged ⩾15 years from across England based on a multistage household sampling	1. Qualitative part:<br>Structed interview explores the recent RTI illness episode and what healthcare advice they had sought, if any.<br>2. Quantitative part:<br>questions about their expectations for antibiotics, and their antibiotic use, covering:<br>1) if they expected their GP or nurse to prescribe antibiotics if they went to see them with different RTI conditions;<br>2) if in the past year they had asked their GP or nurse for antibiotics for themselves or for someone else for any condition and what happened if they asked;<br>3) if in the past year they had been prescribed antibiotics for any condition and if they had finished the course as prescribed, or offered a delayed antibiotic prescription.<br>4) whether they agreed with a number of statements about antibiotics, whether they expected different RTI symptoms would get better more quickly with antibiotics, and how much of a problem they thought antibiotic side effects were.<br>5) whether they had had sore throat, cold, cough, or flu symptoms in the previous 6 months, how the symptoms of their most recent RTI had affected their general health and what actions they took as a result.<br>6) If they had contacted or visited their GP surgery with their most recent RTI they were asked their reasons and expectations for consulting.	Thematic analysis and mean comparison methods (adjusted for demographics)	15/17
Kai, J.,et al. (1996)	Europe	U.K	Parents or caregivers	Qualitative	To identify and explore difficulties parents experience with acute illness in young children and the information they seek to help them.	95 parents of preschool children under 5 years old<br>（Purposeful sampling）	The interviews were open ended, semi-structured. The interviews explored parents' experiences and difficulties coping with ill young children.	Grounded theory methodology and manifest content analysis	9/10
Halfvarsson, J., et al. (2000)	Asia	Viet Nam	Parents or caregivers	Mixed	To investigate rural mothers' perceptions of antibiotics in their use against ARI among children 5 years and under using a combination of	1) 3 key informant interviews with healthcare providers;<br>2) 6 focus group discussions (FGD) covering a total of 46 mothers with children 5-years-old and under (random sampling),<br>3) 185 structured household interviews of	6 Focus groups discussion with mothers cover topics relating to the aetiology of ARI, the prevailing treatment options, treatment periods, and antibiotics, including their negative effects<br>No details about other survey methods;	Descriptive and mean comparison analysis	10/17

					qualitative and quantitative methods.	mothers (SHI),<br>4) 18 semi-structured drug vendor interviews (SSDVI).			
Neill, S. J.,et al. (2010)	Europe	U.K	Parents or caregivers	Qualitative	to discover the psychosocial processes which take place in families when a child is acutely ill at home, and the influence of these processes on families' responses to such episodes of illness.	15 families with children aged 0–9 years (mixed sampling methods: purposive/theoretical/selective sampling)	An un-structured in-depth interviewing explore the family beliefs and experiences:<br>1. starter question: 'What was it like when . . . was ill the other day/last week?'<br>2. Neutral prompts and probes where then used to help research participants to tell more about their experiences, giving the necessary depth to the data<br>3. A draw, write and/or tell technique was used with children over 5 years of age.	Glaserian grounded theory methodology	8/10
Kai, J.,et al. (1996)	Europe	U.K	Parents or caregivers	Qualitative	To identify and explore parents' concerns when young children become acutely ill.	95 parents of preschool children under 5 years old<br>（Purposeful sampling）	The interviews were open ended, semi-structured. The interviews explored parents' experiences and difficulties coping with ill young children.	Grounded theory methodology and manifest content analysis	9/10
Goggin, K.et al. (2020)	North America	U.S.A	Parents or caregivers	Quantitative	To assess the impact of a 90-second animated video on parents' interest in receiving an antibiotic for their child.	English and Spanish speaking parents (n = 1051) of children ages 1-5 years presenting with acute respiratory tract infection symptoms (eg, cough, congestion, sore throat, earache) (Sampling based on a RCT design)	Questionnaire survey.<br>1. The main outcome: parents' interest in receiving an antibiotic for their child (before and after meeting with their provider), using a visual analogue scale ranging from 0 to 100, with 0 being "I definitely do not want an antibiotic," 50 "Neutral," and 100 "I absolutely want an antibiotic."<br>2. Other collected factors: antibiotic knowledge questions (answered 6 true/false); demographics; parent satisfaction.	Bivariate logistic regression analysis	7/8
Emslie, M.J.,et al. (2003)	Europe	Scotland	General population	Quantitative	To gather information on the current state of public knowledge of, and attitudes and behavior to antibiotics	351 patients (age 16-74) from one urban and one rural general medical practices. (Institutions are purposive sampling, patients are systematic sampling)	Questionnaire survey assessing (5-likert scale):<br>1) opinions about antibiotic use;<br>2) sources of advice and information on management of RTI;<br>3) opinions about antibiotic resistance.	Descriptive and mean comparison analysis	4/8
Gaarslev, C.,et al. (2016)	Oceanica	Australia	General population & Parents or caregivers	Mixed	To understand the characteristics of patients who expect antibiotics for an URTI and the underlying reasons for this expectation.	Quantitative part:<br>1509 Australian consumers aged 16+ years were selected proportionate to geographic location (random sampling).<br>Qualitative part:<br>21 people within five groups that were more likely to ask for antibiotics:<br>1) mothers with young children under six years of age;<br>2) low socioeconomic group;<br>3) long term migrants who speak either Arabic or Chinese at home<br>(purposive sampling-inclusion criteria: participants had been prescribed antibiotics for themselves or their children in the last 6 months by their general practitioner (GP).	Quantitative part-questionnaire survey, covering:<br>1) Demographics;<br>2) Medicines respondents take;<br>3) Knowledge, beliefs and expectations for an antibiotic;<br>4) Outcome: self-reported expectation for an antibiotic from a doctor if presenting with a cold or flu.<br>Qualitative part-semi-structured focus group discussion, guideline covering:<br>1) the social and cultural norms surrounding their expectations for antibiotics;<br>2) understanding possible communication strategies to decrease patient demand.	Quantitative part: Regression analysis;<br>Qualitative part: Thematic analysis	14/17

Borg, M.A. ,et al. (2011)	Europe	26 Countries from Europe	General population	Quantitative	To assess whether culture influence individual antibiotic use for colds/flu/sore throat.	26259 responses within 26 countries from Europe<br>(The survey covered residents aged ⩾15 years in each country. Within each country, a multistage, random sampling was applied proportionally to the population size)	Outcome indicator-Summarized questionnaire survey results: Proportions of respondents, by country, who stated that they had taken at least one course of antibiotics in the previous year for colds/flu/sore throat (CFSt).<br>Independent variables-Hofstede's model of cultural dimensions of countries;<br>Individual scores for PDI (Power distance), IND (Individualism), MAS (Masculinity), UAI (Uncertainty avoidance), LTO (Long-term orientation) and IVR (Indulgence) were accessed for each EU country from website.	Multiple linear regression models	6/8
Faber, M.S.,et al. (2010)	Europe	German	General population	Quantitative	To explore knowledge, attitude and expectations of Germany's general public	1,076 persons between the age of 15 and 78 years<br>(general populations who are Internet users, sampling based on populations who are registered with a large market research company)	Online questionnaire survey (multiple-choice, Likert scale) :<br>1) expectations of prescription of antibiotics from physicians;<br>2) knowledge and attitudes regarding effectiveness of antibiotics;<br>3) antibiotic use for upper respiratory tract infections.	Logistic regression analysis	5/7
Davis, M.E.,et al. (2017)	North America	U.S.A	General population	Mixed	1) To explore beliefs about antibiotics and knowledge and awareness of the appropriate use of antibiotics and antibiotic resistance among patients in primary care settings;<br>2) To examine how their beliefs, knowledge, and awareness may be related to their expectations regarding the use of antibiotics in their treatment.	Quantitative part:<br>190 adult patients aged 18 or older at seven primary care clinics and two urgent care locations (convenience sampling).<br>Qualitative part:<br>4 persons from quantitative survey (all were female, had college level education, and were between 25 and 45 years old).	Quantitative part:<br>A 16-item paper-based survey:<br>1) patient knowledge of the appropriate use of antibiotics;<br>2) patient awareness of antibiotic resistance;<br>3) patient perceptions of the outcomes of antibiotic resistance;<br>4) patient expectations regarding the prescribing of antibiotics by providers when they have a cough and common cold;<br>5) patient preferences for treatment (what their provider could do to make them feel better when an antibiotic was not prescribed to treat their illness)<br>Qualitative part:<br>In-depth interview and guideline covers: beliefs about antibiotics, knowledge of the appropriate use of antibiotics, awareness of antibiotic resistance, expectations regarding the use of antibiotics in their treatment, and preferences regarding what providers could do to make them feel better if they were not given a prescription for an antibiotic	Regression analysis and thematic analysis	12/17
Kong, L.S.,et al. (2019)	Asia	Malaysia	General population	Quantitative	1) To assess older adult population knowledge on antibiotic use and resistance and their expectations towards the need for antibiotics.<br>2) To assess the relationship between knowledge, expectations and practices related to antibiotic use among older adults.	402 Malaysian citizens aged 60 years and above.<br>(No details about sampling, seemed convenience sampling)	A questionnaire survey based on other validated instrument, covering four parts:<br>1) demographic characteristics;<br>2) respondents' prior experience of antibiotic use and inappropriate practices regarding antibiotic use;<br>3) knowledge on antibiotic use and knowledge on antibiotic resistance;<br>4) respondents' expectations towards antibiotic use, including respondents' expectations to be prescribed with antibiotics for selected symptoms (7 statements) and respondents' expectations from the physician in relation to antibiotic prescribing.	Descriptive and mean comparison analysis	4/8

Mainous, A.G., et al. (1997)	North America	U.S.A	General population	Quantitative	to examine patient knowledge of the normal presentation of URIs, patient expectations for health services, and patient belief in the effectiveness of antibiotics as a treatment modality.	961 persons:<br>An undifferentiated patient population who are adults (18 years of age or older) based on four different sites (mainly healthcare facilities but also include community facility)	A questionnaire survey based on two illness scenarios (normal presentations of an uncomplicated URI), covering three aspects:<br>1) knowledge of the normal presentation o f a URI,<br>2) effectiveness of treatments,<br>3) health care utilization.<br>Two illness scenarios are:<br>A. "You have had an illness for five days with the following symptoms: sore throat, cough, and runny nose with clear discharge";<br>B. "You have had an illness for five days with the following symptoms: sore throat, cough, and runny nose with discolored discharge (yellow, green, brown)."	Regression analysis	6/7
Faidah, H.S.,et al. (2019)	Asia	Saudi Arabia	Parents or caregivers	Quantitative	To analyze parental knowledge, behavior and perception towards the antibiotic consumption in the treatment of URTIs in children.	570 parents of students from Kindergarten (5 years) and first-year students (6 years) (Cluster sampling)	A 23- item self-administered questionnaire, covering:<br>1) Demographic characteristics of the respondents.<br>2) Knowledge about antibiotics and Upper respiratory tract infections and their attitude towards using antibiotics for upper respiratory tract infections.<br>3) parents approach and expectations from pediatricians for prescribing antibiotics to their children suffering from URTIs, and their attitude towards using antibiotics without pediatrician advice and factors affecting this attitude.	regression analysis	5/8
El Khoury, G.,et al. (2017)	Asia	Lebanon	Parents or caregivers	Quantitative	To target parents, as primary caregivers, to assess their knowledge and practices toward antimicrobial agents administered to their children for URTIs.	1,037 participants who have children between 2-12 years old.<br>(No specific inclusion and exclusion criteria were presented, convenience sampling)	Questionnaire survey covers:<br>(1) parents' sociodemographics characteristics;<br>(2) general information about children's health;<br>(3) parents' perspective regarding antimicrobial resistance and their knowledge on antibiotics use for URTIs;<br>(4) parents' practices toward antibiotic uses for URTI:<br>(5) their source of information on antibiotics.	regression analysis	6/8
Cornford, C.S.,et al. (1993)	Europe	U.K	Parents or caregivers	Qualitative	To examine mothers' personal accounts of their concerns about their child's cough, their perceptions of the benefits in consulting and to evaluate how they assess their child's illness.	30 mothers of children to 5 years old whose main symptom was a cough and who had decided to consult a GP.	No details of the guideline of interviews.<br>Interviews are based on a schedule of open questions that focused on broad areas they wished to explore based on themes identified in a pilot study.	No details	6/10
Lanyero, H.,et al. (2020)	Africa	Uganda	Parents or caregivers	Quantitative	To investigate prevalence of, and factors associated with, use of antibacterials in management of symptoms of ARIs in children under five years in rural communities of Gulu district, northern Uganda.	856 child care-givers in households with children under five years who had displayed symptoms of ARIs within two weeks preceding the data collection date (Multi-stage sampling: purposive sampling in cluster and probability proportionate to size (PPS) used)	A questionnaire survey, covering:<br>Demographics, symptoms of disease, medication given and the source of the medicine used, information on the medication they used, and whether the medicines were used as a result of self-medication or prescribed by a health care professional.	regression analysis	5/8

You, J.H.,et al. (2008)	Asia	Hong Kong, China	General population	Quantitative	To examine public knowledge, attitudes and behavior regarding antibiotic use in the community of Hong Kong.	1,002 non-institutionalized Hong Kong residents.<br>(Random sampling)	A phone survey based on interview. Questions covers:<br>1) Knowledge and attitudes regarding appropriate antibiotic use for treatment of general symptoms (sore throat and runny nose) of upper respiratory tract infections (URTIs) and symptoms associated with more serious URTIs (sore throat, runny nose, cough and fever).<br>2) Knowledge on the indications for antibiotics, compliance to completing the prescribed course of antibiotic and risk factors related to bacterial resistance to antibiotic therapy.<br>3) Participants' most recent episode of URTI treated by antibiotics and the types of medical service received;<br>4) Demographics	regression analysis	5/8
Kandeel, A.,et al. (2014)	Africa	Egypt	General population	Mixed	To explore the knowledge, attitudes, and practices of patients regarding antibiotic use for ARIs, and to identify cultural and societal determinants contributing to the use of antibiotics in Egypt	Quantitative part:<br>350 Patients with a physician diagnosis of common cold, otitis media, sinusitis, bronchitis, pharyngitis, influenza, or pneumonia; (123 Adults and 227 children, parents/caregivers as representers).<br>(Seemed cluster random sampling, but the recruit of participant in each facility seemed not randomized)<br>Qualitative part:<br>20 focus group discussions (FGDs) including 160 participants, equally divided into women or men with higher or lower education levels.	Quantitative part:<br>Questionnaire survey, covering<br>Demographic characteristics, presenting symptoms, and characteristics of the current illness, type and dosage of antibiotics prescribed, patient knowledge, attitudes, and beliefs regarding antibiotic use.<br>Qualitative part:<br>Focus group discussion. No details about discussion guideline.	Mean comparison analysis and thematic analysis	11/17
Hernández-Díaz, I.,et al. (2019)	North America	U.S.A	Parents or caregivers	Quantitative	To evaluate Latino parents or legal guardian knowledge and beliefs, behaviors, and adherence regarding antibiotic use for URTIs in their children younger than 6 years of age.	101 parents or legal guardians of at least one child younger than 6 years<br>(Convenience sampling, recruited from emergency departments with a pediatric unit)	A questionnaire survey, covering 3 parts:<br>(1) knowledge and beliefs (parents' knowledge and beliefs regarding antibiotic use in their children's URTI),<br>(2) behaviors (parents' behaviors regarding antibiotic use in their children for URTI),<br>(3) adherence (parents' expected adherence to antibiotic treatment regimens to treat their children with URTI, including dosage, frequency,<br>omission of therapy, and duration/completion of antibiotic therapy).	regression analysis	5/8
Freidoony, L.,et al. (2017)	Asia	Korea	General population	Quantitative	To develop a description of the components of the 'RTI clinical iceberg' by exploring the practices, expectations, and adherence of the general public toward RTIs and antibiotics.	547 general public visiting Wonju Severance Christian Hospital (aged >= 18)<br>(Convenience sampling)	A questionnaire survey, covering:<br>1) Practice of antibiotic use;<br>2) Knowledge and attitudes towards antibiotic use and antibiotic resistance;<br>3) Management of RTIs;<br>4) Demographics;	Descriptive and mean comparison analysis	4/8
Ngu, R.C.,et al. (2018)	Africa	Cameroon	General population	Quantitative	To provide data on the prevalence of antibiotic self-medication in adult patients with RTI and identify the factors	308 Patients aged 21 years and above who presented with symptoms of RTI<br>(Consecutive included, seemed convenience sampling)	A questionnaire survey, covering:<br>1) demographic characteristics,<br>2) self-medication use,<br>3) sources of self-medication<br>4) signs or symptoms of respiratory tract infections	Descriptive and mean comparison analysis	5/8

					influencing this practice in an urban hospital in Cameroon.			
Rönnerstrand, B.,et al. (2015)	Europe	Sweden	General population	Quantitative	1) To investigate the influence of reciprocity on the willingness to postpone antibiotic treatment in order to avoid unnecessary use;<br>2) To investigate the association between generalized trust and the willingness to postpone antibiotic treatment for the same reason.	981 individuals from Swedish citizens (randomly drawn from an electronic panel of Swedish citizens)	A hypothetical scenario experimental design with scenario vignettes used (background factors, generalized trust, trust in health care and demographic and health-related variables were measured also):<br>1) The respondent is visiting a doctor due to a respiratory infection. The doctor prescribes antibiotics, but recommends postponing antibiotic treatment in order to see if the disease resolves by itself, for the sake of avoiding unnecessary medication.<br>2) The doctor says that reason for postponing treatment with antibiotics is that unnecessary antibiotic therapy contributes to bacteria becoming resistant to antibiotics. The physician also says that antibiotic resistance is a societal problem that compromises the treatment of infections where antibiotics is necessary and complicates operations, transplants and cancer treatment.<br>3) The doctor also says that about two out of three (67%) of all patients wait at least [1/3/5] day[s] before beginning antibiotic treatment. Scenario stimuli manipulation is in square brackets, implying that the number of days most patients postpone treatment varies between one, three, and five days, respectively. In the control scenario, there is no information at all about the number of days most people accept postponing treatment.<br>4) Subsequent to the hypothetical scenarios respondents are asked to assess their own willingness to postpone antibiotic treatment. In doing so, the respondents indicate the number of days that they would accept postponing antibiotic treatment. This was done on a scale ranging from 0 to 7 days.	Descriptive and mean comparison analysis	4/7
Wilson, A.A.,et al. (1999)	North America	U.S.A	General population	Quantitative	To describe experiences and beliefs regarding antibiotic effectiveness, and to evaluate the association between experiences, beliefs, and use of antibiotics for ARIs.	386 Adults aged 18 years or older (Random sampling based on a computer-assisted telephone survey)	Telephone interviews, covering:<br>1) knowledge of antibiotic effectiveness;<br>2) use of antibiotics for respiratory illness;<br>3) experiences with advice seeking and with the health care system,<br>4) sociodemographic information,<br>5) Other factors (to control confoundings)<br>5.1 whether the participants had children aged 5 years or younger (hypothesizing that these persons are more likely to contacting healthcare system regarding antibiotic treatment)<br>5.2 antibiotic belief;<br>5.3 antibiotic experiences;<br>5.4 chronic conditions;	Regression analysis	4/8

Grigoryan, L.,et al. (2007)	Europe	19 Europe countries	General population	Quantitative	To investigate whether prescribed use triggers self-medication with antibiotics in European countries.	15,548 Adult randomly selected from 19 European countries<br>(A multistage sampling)	Questionnaire cover questions:<br>1) the respondent's use of antibiotics during the past 12 months,<br>2) name of the medicine, source,<br>3) symptom or disease (coded with ICPC10)<br>4) demographic characteristics.	Regression analysis	5/8
Jakupi, A.,et al. (2019)	Europe	Kosovo	General population	Qualitative	To explore the attitudes, experiences and knowledge of users, pharmacists and prescribers towards antibiotics in Kosovo.	Four groups of people:<br>1) 4 Patients who recently received a prescription for one of the antibiotics (snowballing sampling);<br>2) 4 Patients who recently received one of the antibiotics without a prescription (snowballing sampling);<br>3) 4 Community pharmacists (purposive sampling);<br>4) 4 Physicians with recent experience of dispensing or prescribing antibiotics (purposive sampling)	The semi-structured interviews are based on a interview guideline, focusing on certain antibiotics for an upper respiratory tract infection (URTI).(Four commonly used antibiotics were chosen, i.e., amoxicillin-clavulanic acid, azithromycin, ciprofloxacin or ceftriazone,)	Deductive or directed content analysis	10/10
Mangione-Smith, R.,et al. (2004)	North America	U.S.A	Parents or caregivers	Quantitative	To determine whether parent expectations for antibiotics vary by race and ethnicity	543 Parents with child having URI symptoms (aged from 6 months to 10 years) and without antibiotics during the prior 2 weeks<br>(Cluster sampling, seemed convenience sampling in each cluster - physician level)	Parents completed a self-administered previsit questionnaire that collected information on their:<br>1) demographics,<br>2) their expectations for the visit,<br>3) their child's current symptoms,<br>4) the duration of their child's illness in days,<br>5) their level of worry about their child's illness,<br>6) whether their child attended day care or preschool,<br>7) whether the physician being seen was their child's primary medical doctor, and if so, for how long.	Regression analysis	8/8
Roberts, R.M.,et al. (2015)	North America	U.S.A	General population & Parents or caregivers	Qualitative	To understand participants' knowledge and attitudes regarding antibiotic-associated ADEs.	45 participants (adults and mothers of young children), including:<br>1) 22 adults aged from 25 to 53 years;<br>2) 23 mothers who had children aged 2 to 11 years<br>(Recruited by a professional recruitment firm based on a national database, seemed random sampling)	6 focus group discussion about participants' knowledge and attitudes regarding antibiotic resistance and use as well as ADEs associated with antibiotic use, including:<br>1) 3 group discussion with Mothers, covering their experience of URIs and use of antibiotics for their children.<br>2) 3 group discussion with adults, covering their personal experiences with URIs and antibiotics.	Grounded theory and thematic analysis	7/10
Hassan, M.Z.,et al. (2020)	Asia	Bangladesh	Parents or caregivers	Quantitative	To examine the prevalence and factors associated with antibiotic use in under-5 children with ARIs based on nationally representative population-based sample survey data	2144 children who had an ARI episode within the preceding 2 weeks	Mothers were asked about any recent illness episodes of their under-5 children including any illness with symptoms of cough, fever, runny nose<br>or diarrhoea (results not used) in the 2 weeks preceding the survey with response options 'yes', 'no' or 'don't know'. If answered 'yes', mothers were also asked further details of any treatment sought; including, place of treatment, type of facility and type of drugs used.<br>Other exploratory factors included:<br>1) socio-demographics; 2) Mother and child characteristics; 3) child nutrition status; 4) drug source and providers;	regression analysis	6/8

Parimi, N.,et al. (2004)	North America	Trinidad and Tobago	Parents or caregivers	Quantitative	To investigate the knowledge, beliefs and practices of children's caregivers in Trinidad and Tobago regarding antibiotic utilization and explored these beliefs in self-administration of antibiotics in childhood URTIs	417 general population who had a child ≤ 12 years<br>(randomly selected based on telephone survey)	Self-developed questionnaire, covering:<br>1) Demographics;<br>2) caregivers' knowledge (identification of antibiotics) and beliefs (cue all infections, side effect, safe)<br>3) caregivers' practices of antibiotic use (symptoms which children in their care had in the past 30 days, their assessment of the symptom severity, whether they sought medical assistance and if they administered any antibiotic to the child.)	Descriptive and mean comparison analysis	4/8
Landers, T.F.,et al. (2010)	North America	U.S.A	General population	Quantitative	1) To assess the extent to which antibiotics and nonantibiotics commonly used for upper respiratory infections (URIs) were correctly recognized by a sample of urban-dwelling Latinas;<br>2) To determine if correct classification by respondents was associated with antibiotic use within the household or self-medication with antibiotics	100 Latino households randomly selected from a clinical trial (criteria: households included at least three persons with at least one child under 5 years), covering<br>1) 50 households in which participants initially reported antibiotic use;<br>2) 50 households, which did not initially report antibiotic use.	Home visits and face-to-face interview questionnaire:<br>1) Whether any member of their household took medications for URI symptoms and to identify the name of each medication;<br>2) Age of household member who taken antibiotics (if taken);<br>3) where the antibiotics were obtained;	regression analysis	5/8
Friedman, J.F.,et al. (2003)	North America	U.S.A	Parents or caregivers	Quantitative	1) To describe URI and antibiotic knowledge, beliefs about day care policies regarding URIs;<br>2) To identify both parent and day care center predictors that might contribute to parental acute care and antibiotic seeking for children in day care.	Staff survey for Day care center directors and lead teachers;<br>85 staff within 36 days care centers (capacity of 5-75 children and children enrolled younger than 48 months-Randomly selected)<br>Parent survey for parents<br>211 parents in the participating day care centers (child should age from 6 - 48 months)	Dependent variables (from parent survey):<br>1) acute care seeking for upper respiratory symptoms (URS) in the absence of fever and with normal activity level: clear runny nose, green runny nose and cough;<br>2) belief that antibiotics expedite return to day care for the same 3 symptoms;<br>Independent variables (from staff survey and parent survey)<br>1) day care center exclusion and physicians clearance policies (staff)<br>2) day care enforcement of rules for exclusion of ill children (staff);<br>3) parent knowledge and beliefs regarding course of URIs and antibiotic indications for specific URIs (parent)<br>4) attitudes and beliefs about day care center policies (parent)<br>5) demographics (parent);	regression analysis	6/8
Togoobaatar, G.,et al. (2010)	Asia	Mongolia	Parents or caregivers	Quantitative	To determine the prevalence of the administration of non-prescription antibiotics by caregivers to children younger than 5 years of age, and to identify factors associated with non-prescription use.	503 households with more than one child aged < 5 years<br>(a representative sample, two-stage cluster sampling)	Previous validated structured questionnaire, covering:<br>(i) the socioeconomic and demographic characteristics of households;<br>(ii) caregivers' knowledge and attitudes regarding antibiotic use to treat respiratory illnesses as well as about the usefulness of antibiotics for bacterial and viral infections;<br>(iii) the use of antibiotics for the index child in the previous 6 months.<br>(iv) If mothers or caregivers reported that their child had taken antibiotics without a prescription, they were asked for further	regression analysis	6/8

							details concerning self-medication with antibiotics (their reasons for self-medication, describe the symptoms, identify the source, names and dosages of antibiotics, and state the duration of use.).		
Bianco, A.,et al. (2020)	Europe	Italy	General population	Quantitative	1) To investigate the knowledge and practices regarding antibiotics use and AMR among the general population<br>2) To analyze whether sociodemographic characteristics of the population could be associated with poor knowledge and improper practices.	568 patients recruited at randomly selected days from attending 17 general practitioners (GPs) and 10 community-based pediatrician (CBP)	Self-developed questionnaire covered:<br>1) sociodemographic characteristics of the participants;<br>2) knowledge on antibiotics and AMR;<br>3) practices regarding the consumption of antibiotics.	regression analysis	4/8
McKee, M.D.,et al. (1999)	North America	U.S.A	General population	Quantitative	To examine patients' knowledge of URI presentation, belief in the effectiveness of antibiotics, and practices regarding the use of antibiotics for common colds in an ethnically diverse community.	192 Adults with 18 years of age or older from diverse health care settings (outpatient clinics, private practices, emergency departments) and a community center	Survey based on two simulated scenarios (uncomplicated URIs):<br>1) Scenarios presentation:<br>Scenario 1:"You have had an illness for 5 days with the following symptoms: sore throat cough, and runny nose with clear discharge";<br>Scenario 2: "You have had an illness for 5 days with the following symptoms: sore throat, cough, and runny nose with discolored discharge (yellow, green, brown)."<br>2) Simulated question answered:<br>Then, respondents were asked to report their likelihood of seeking care, usual treatments for the illness described, and the effectiveness of a variety of treatments (including antibiotics, randomly assigned options).<br>3) Relevant practices:<br>The subjects were asked if they had ever taken antibiotics that were not prescribed for them by a physician and, if so, how they obtained them.	Descriptive and mean comparison analysis	4/7
Hong, J.S.,et al. (1999)	North America	U.S.A	General population	Quantitative	To test hypothesis that patients' current desires would be shaped by past experiences, such that patients who had received antibiotics in the past would expect them again.	70 Patients who were 18 to 55 years old with a chief complaint of upper respiratory infection-type symptoms (eg, cough, sore throat, runny nose).	Self-developed questionnaire, covering:<br>1) upper respiratory infection symptoms (type, number, and duration),<br>2) demographic characteristics,<br>3) desire for antibiotics<br>4) past experiences seeking care for upper respiratory infections	regression analysis	7/8

Simon, A., et al. (1996)	Asia	Philippines	Parents or caregivers	Qualitative	To document caretakers' actions when children are sick with cough, and describes home management, health-seeking behavior, and attitudes and practices with regard to the administration of pharmaceuticals.	65 mothers and 12 grandmothers presenting with sick children (< 5 years) to the pediatric outpatients clinic of hospital; 45 healthcare providers	Caretakers:<br>1. information regarding caretakers' health seeking practices when their child was sick, their knowledge about antibiotics, and their reasons for using them during an ARI episode.<br>2. Follow-up interviews (n=16) in 5-7 days after the initial interview to monitor the progress of their sick child and their compliance with prescriptions and medicine instructions<br>Healthcare providers:<br>nurses, midwives, barangay health workers, and doctors (N=45) were interviewed to gain a broad understanding of patterns of diagnosis, treatment, prescription and compliance.<br>Informal observation of physician-patient encounter:<br>Concerning the interaction between the mothers and health providers.	No details	6/10
McNee, A., et al. (1995)	Asia	Philippines	Parents or caregivers	Qualitative	To explore the health seeking behaviors of caretakers in response to ARI in children under five years of age	101 Caretakers:<br>Caretakers with children less than five years old (purposive sampling based on a recent 3-6 month episode of ARI).	Nine focus groups (average 7 per group) and 42 in-depth interviews<br>Caretakers' focus group and interviews guides was developed as following:<br>Caretakers' children's recent episode of ARI episode was used to form the basis for discussion in focus groups and in-depth interviews, aiming to detail the factors that influenced behavior around a specific event, and to contrast it with other episodes and respondents' general views.	A phased iterative research process	5/10
Gu, J., et al. (2015)	Asia	China	General population	Quantitative	To investigate the differences between rural and urban residents with regard to antibiotic knowledge and use and to review the factors that were associated with the knowledge of, attitude towards and use of antibiotics in this population.	3631 subjects over the age of 18 (randomly recruited)	Self-developed questionnaire, covering:<br>1) demographic characteristics;<br>2) knowledge of the use of antibiotics;<br>3) attitude towards antibiotic use;<br>4) personal use of antibiotics;	regression analysis	4/8
Juma, M. et al. (2016)	Asia	Oman	General population	Qualitative	To explore the Omani public's knowledge and attitudes to the use of antibiotics for the common cold and to understand cultural factors that could contribute to AMR.	Twenty-one consented participants (convenience, purposive and snowball sampling sampling)	A semi-structured interview, Guidelines cover:<br>the views and the attitudes of the public toward self-care and management of minor ailments, including the common cold	Constructivist grounded theory	8/10
Mortazhejri, S. et al. (2020)	North America	Canada	General population	Qualitative	To explore how individuals perceived URTIs and how their perceptions influenced their management of URTIs (including self-management, primary care	15 adult people who had have previous experience of having a URTI	A semi-structured interview, interview guide cover:<br>Demographic questions about gender, age and level of education, as well as openended questions related to individuals' experiences with URTIs	Thematic analysis	8/10

					consultation and antibiotic use)				
Pechere, J.C. (2001)	Multi-region	Asia, Africa, and Colombia	General population & Parents or caregivers	Quantitative	To learn more about patients' perceptions of common respiratory tract infections, their requirements for antibiotic use, and their adherence to the treatment as prescribed.	5379 people who had either taken a course of antibiotics or had given one to their child for an ambulatory respiratory tract infection from 9 countries<br>(Randomly digital dialing)	21 multiple choice questions: covering:<br>1) socioeconomic status<br>2) the seriousness of the respiratory tract infection for which the antibiotic was prescribed<br>3) how the patient or parent viewed the need for medical advice and drug therapy.<br>4) belief in antibiotic therapy, the way the drugs work and their effects, and<br>their perception of doctors<br>5) adherence to their most recent course of antibiotics in terms of taking the required number of daily doses and the full duration of the course.	Descriptive analysis	5/6
Larsson, M. (2000)	Asia	Viet Nam	Parents or caregivers	Quantitative	To investigate antibiotic use and antibiotic susceptibility of respiratory tract pathogens in children aged 1–5 years in Bavi, Vietnam	166 carers of 200 children aged 1-5<br>(Random sampling from an established cohort)	Questionnaire survey, covering:<br>1) what type of antibiotic was used, how long treatment lasted<br>2) where the antibiotics had been purchased<br>3) what type of treatment information carers had retained	Descriptive analysis	5/6
Kaplan, NM., et al.(2020)	Asia	Jordan	General population	Quantitative	To explore the association of several socioeconomic factors with changes in antibiotic request by the patients following TAP intervention	855 subjects (506 in the control group and 349 in the intervention group)	Self-developed questionnaire to assess the demand for antibiotics	Descriptive and mean comparison analysis	4/8
vanovska, V., et al.(2013)	Europe	The Republic of Macedonia	General population	Quantitative	To assess public knowledge, beliefs and behavior regarding antibiotic use in adults and children, with special interest on URTI	Adults aged 18 and older, who, where relevant, were asked to provide information on their children aged 0–4 years	Self-developed questionnaire:<br>1) participants' demographic and socioeconomic characteristics;<br>2) knowledge and beliefs regarding antibiotic use to treat upper respiratory infections;<br>3) sources of oral antibiotics obtained in the past year;<br>4) actual behavior during last URTI episode in the six months preceding the study	regression analysis	4/8
Chao DVK., et al.(2004)	Asia	Hong Kong, China	General population & Parents or caregivers	Quantitative	To explore the URTI-related knowledge, consultation behavior, self-care practice and the reasons for consultation at GOPC.<br>To explore determinants of URTI-related knowledge, self-care practice and consultation behaviors.	443 guardians and 448 adult patients	Self-developed questionnaire, no detail.	mean comparison and regression analysis	7/8

Tan YSL., et al.(2006)	Asia	Singapore	General population	Quantitative	To study the knowledge factors influencing both treatment-seeking behavior and antibiotic cognition in adult patients seeking consultation for upper respiratory tract symptoms in all nine polyclinics in the National Healthcare Group, Singapore	Aged 21 years and above, who complained of any one of the following symptoms	Self-developed questionnaire, questionnaire covers:<br>1) participant's personal and demographical data<br>2) his knowledge about URTI<br>3) past experiences with URTI<br>4) treatment-seeking behavior for a current episode of URTI, including reasons for his attendance	Regression analysis	5/8
Chlabicz, S., et al.(2018)	Europe	Poland	General population	Quantitative	To describe how frequently patients presenting with RTIs to their primary care provider openly express their wish not to be treated with antibiotics, which symptoms and physical findings were related to their perception of antibiotics not being helpful, to what degree their expectations influenced doctors' decisions, and what facts were used to present their viewpoint.	1456 patients with RTIs	Direct observation study, focusing:<br>patients' symptoms, physical examination findings, diagnoses and the doctors' decisions to prescribe antibiotics	Regression analysis	5/8
S. Kaae, et al (2017)	Europe	Albania	General population	Qualitative	To investigate the AB knowledge, attitudes and behaviors of patients and HCPs	16 participants cover four types of populations via convenience sampling (each of four):<br>1) patients/adults who had used ABs with a prescription in the past 3 months<br>2) patients/adults who had used/purchased ABs illegally without a prescription in the past 3 months<br>3) Community pharmacists (both admitting to legal and non-legal sales of ABs)<br>4) Physicians working in the primary care system	Semi-structured interview covers seven questions:<br>1) the process of diagnosis<br>2) how and why a specific AB was selected<br>3) where and how ABs were purchased<br>4) the patients' use of ABs<br>5) satisfaction with the AB process<br>6) AB knowledge<br>7) AB attitudes	Theoretical driven thematic analysis	9/10
E. A. Belongia., et al (2002)	North America	U.S.A	General population & Parents or caregivers	Quantitative	To collect baseline information on public knowledge, attitudes, and experiences regarding appropriate antibiotic use	405 adults and 275 parents of children aged < 5 years old<br>(Random-digit-dialing sampling)	Telephone survey using structured interview, covering:<br>1) attitudes and beliefs regarding appropriate indications for antibiotic use to treat respiratory illness<br>2) exposure to informational messages about antibiotic-resistant infections<br>3) respondents who had seen a physician during the past 6 months for acute respiratory illness were asked about<br>3.1) their expectations and experiences<br>3.2) level of satisfaction with the visit<br>4) antibiotic knowledge	Regression analysis	5/8

M. Bakhit, et al (2019)	Oceanica	Australia	General population & Parents or caregivers	Qualitative	To explore patients' and parents' understanding of antibiotic resistance and implications for decisions about antibiotic use	32 patients or parents of child patients presenting to general practice with an ARI. (convenience sampling)	Interviews guides includes:<br>1) 'Usual' approaches of expecting and/or using antibiotics for managing<br>acute respiratory infections<br>2) Understanding of the meaning of 'antibiotic resistance', its cause/s,<br>and implications of it<br>3) Awareness that antibiotic resistance can spread between those in<br>close proximity<br>4) Awareness that antibiotic resistance can decay over time	Thematic analysis	8/10
S. M. Hussain, et al (2020)	Asia	Saudi Arabia	Parents or caregivers	Quantitative	To explore the awareness of parents of children younger than 5 years with regard to management of childhood fever	1700 parents of children younger than 5 years with regard to management of childhood fever	Questionnaire survey covers:<br>1) demographics<br>2) knowledge<br>3) attitudes<br>4) practices	Regression analysis	5/8
Y. Luo, et al (2021)	Asia	China	General population	Quantitative	To investigate the non-prescription antibiotic use for cough in China and explore to which extent antibiotic use knowledge was correctly instructed in communities.	3034 respondents who aged 15 years or older<br>(Random sampling)	Questionnaire survey covers:<br>(1) sociodemographic information;<br>(2) non-prescription antibiotic behaviors during cough;<br>(3) antibiotic knowledge<br>(4) sources and reasons of antibiotic use when they cough	Regression analysis	7/8
D. Osborne and H. Sinclair (2006)	Europe	U.K	General population	Quantitative	To compare patient knowledge, attitudes and behavior regarding antibiotic use between 2000 and 2005	248 patients (aged 16–74 years)<br>(Institutions are purposive sampling, patients are systematic sampling)	Questionnaire survey assessing (5-likert scale):<br>1) opinions about antibiotic use;<br>2) sources of advice and information on management of RTI;	Descriptive and mean comparison analysis	4/8
L. S. J. Roope, et al (2020)	Europe	U.K	General population	Quantitative	To test the likely impact of fear-based messages, with and without empowering self-efficacy elements, on patient consultations/antibiotic requests for influenza-like illnesses, using a randomized design	4000 adults in the UK<br>(Via Survey Sampling International, claimed to generate a representative sample of UK adult population in terms of sex, age, ethnicity and geographic region)	A randomized experiment based on online questionnaire survey:<br>1) Randomized experiment design:<br>1.1) Simulation of a influenza-like-illness as 'a temperature, aching muscles, a headache, a dry chesty cough, a sore throat, and you feel weak'<br>1.2) Respondents randomized into three groups to receive different messages about antibiotics and AMR, with Group 1 of "fear" message only, Group 2 of "mild fear plus empowerment" message and Group 3 of "strong fear plus empowerment" message<br>2) Measurements<br>2.1) Socio-economic factors<br>2.2) 'To what extent is this information new to you?' [very new/somewhat new/not very new/not at all new]<br>2.3) 'How will this information affect whether you visit a doctor the next time you have symptoms like Health State A?' [much more likely to visit/more likely to visit/no effect/less likely to visit/ much less likely to visit/do not know]<br>2.4) 'How will this information affect the likelihood of you asking a doctor for antibiotics if you were to visit for these	Descriptive, Wilcoxon's rank-sum tests and regression analysis	6/7

		symptoms?' [much more likely/more likely/no effect/less likely/much less likely/do not know]

Supplementary file S4 - Stages, themes and example quotes based on qualitative synthesis

Stages	Themes	Quotes
Need recognition	Symptom recognition	•Parents were generally confident about recognizing whether their child was becoming ill. They used combinations of behavioral and symptomatic clues assessed against any deviation from what was normal for their particular child. [41]<br>•When asked about their symptoms of URTIs, participants often described cough, runny or stuffed nose, post-nasal drip, sore throat, headaches and body aches. [94]
	Cause attribution	•There are two important principles underlying this folk classification of illness-misfortune': (1) the relation of man with nature, i.e. with the natural environment, in Colds and Chills, and (2) the relation of man to man, which exists within human society, in Fevers. [22]<br>•Some participants included both in their answers, suggesting that these are not mutually exclusive views. People get the flu by contacting it from other people who have it. It is caused by a germ that they have and coughing spreads it. It can also result from being over exposed to the cold. [2]<br>•The other account stressed the contagiousness of colds and suggested that they were caused by microbes (ie, bacteria or viruses). [2]<br>•From the FGDs it was evident that the mothers attributed the cause of ARI to changes in weather or cold weather. Only one mother mentioned bacteria as a cause and none mentioned virus.[42]
	Illness labelling	•Two broad categories of illness were identified: minor/normal illness for people generally or for children and/or for the individual child; and 'real illness' requiring medical help. [43]<br>•Women regarded flu and colds as a "disease for everyone", which could be managed at home and did not usually interfere with everyday activities. [7]<br>•In both communities mothers recognized several different illnesses which involve ARI signs and symptoms. Mothers talked about these as distinct illnesses, but also as different levels of severity on a continuum of illness. Mild illnesses could turn into one of the more severe illnesses if not cared for properly. [27]<br>•In relation to cough, they expressed uncertainty about the way to distinguish between a "normal" cough which would be self-limiting and a cough which needed to be seen and treated by a doctor. [32]<br>•Parents seemed to define a child's illness as real when the illness is unknown to them, when it persists or severity increases, when symptoms recur or when symptoms of concern are identified such as a rash.[43]<br>•They talked about both the severity of symptoms (how chesty the cough / how high the temperature) and the duration of symptoms (how long should a cough go on for before consulting the doctor)...[32]<br>•The perceived threat of a cough to a child included a combination of the severity of the illness and the susceptibility of a particular child to developing a cough.[32]
Information seeking	Authenticity	***HCPs:***<br>•Parents described how they were unable to obtain helpful information or advice to facilitate their decision to consult. [32]<br>•When asked about sources of information, answers typically referenced family, physicians, pharmacists, the internet, patient information leaflets, magazines or experience. A television campaign from the Kosovo medical agency was also mentioned by a few of the patient respondents. [72]<br>•Parents drew on family, friends, or the local pharmacy for guidance but usually consulted, or were advised to consult, their doctor when an illness was causing them concern. [41]<br>•There are so many people that are not medically educated . . . I think that's something that we should leave up to the doctor . . . because we didn't go to school for that... I think a lot of people don't know when they really need it (antibiotics). [50]<br>•In this study, participants reported that taking antibiotics for flu was common in their com- munity and that health professionals in Samoa routinely prescribed antibiotics for flu. This suggests that the community has learned from health professionals that antibiotics are the appropriate and responsible ways to treat flu. [2]<br>•Contradictory information or advice was likely to contribute to a decision to consult. [32]<br>•Much health education and the advice given by providers to patients was pragmatic: adhering to timing and completing the course; not sharing prescribed medicine with others; not using anybody else's medicine. [7]<br>•Participants thought that they received insufficient information or explanation from their general practitioner about what was wrong. The most common example was a child being given a diagnosis of a viral or self-limiting illness. Being told their child had a virus or bug was often confusing, provoking anxiety rather than reassurance and sometimes anger. [41]<br>•Some doctors push antibiotics all the time, some refuse to give it. I don't really understand the reasons. They always say it's viral. [47]<br>•Apparent variation in doctors' decisions about prescribing antibiotics created confusion for parents, frustrating their ability to make sense of the illness and learn for the future. [41]<br>***Personal experience:***<br>•Participants expressed valuing their own personal knowledge of how they and their family members experience ARIs and how best to manage these symptoms.[28]<br>•The attitude of the public in managing common colds is influenced by their knowledge and beliefs about the need for antibiotics for full and fast recovery from the symptoms. This attitude was found to be driven by patients' previous treatments obtained for the common cold that influenced their decision when the symptoms recurred. [94]<br>•The adults did not believe that the potential for ADEs was a significant issue, and most reported never discussing ADEs with their provider. They reported relying on their providers' professional judgment as well as their own experiences with using antibiotics in the past to determine whether antibiotics were necessary. [74]<br>•Claire asked the patient, "How are you doing?" and she said, "Well, I'm coughing up phlegm, I ache and I have chills and a sore throat." Claire said, "You have bronchoconstriction, and 3 times a day, if you need to, you should use proventil." The patient asked if she could have an antibiotic for her cold. [12]<br>•Although some physicians and patients are aware that not every complaint needs to be treated by medicines, many Flemish are accustomed to leaving the consulting room with some medicine or other.[30]<br>***Social network:***<br>•Individuals draw upon familiar and well-rehearsed self-care strategies, and those strategies advised by their network members, to manage what they consider to be. [5]<br>•Special value was placed on information provided by network members who had shared experiences as this sharing added credibility to the advice they gave.[5]<br>•NHS branded websites were generally more trusted than other internet sources, as were people in their social network with some health training. [32]<br>•...AMR may not be subject to contagion as it is not discussed within personal networks. [5]

		Other supplementary sources:<br>•The villagers' information about antibiotics also came from media including TV, radio and newspaper [10]<br>•Although many parents suggested TV adverts, websites and social media may be good formats for campaigns, some parents felt these were impersonal without opportunity for dialogue. [8]<br>•One parent recognized that there was contradiction between public health messages that discuss antibiotics. [8]<br>•When viewing campaign material to reduce antibiotic consumption, most parents were happy to avoid antibiotics for simple 'low- level' symptoms they felt comfortable managing at home. [8]
	**Usability**	•Parents thought that sharing common experiences of dealing with illness in young children would be useful in future guidance and make it more relevant, particularly for inexperienced parents. [41]<br>•Parents also wanted information to help them understand and support their management of the illness including signs of serious illness (when do I need to worry?), how to care for child (what might help, what to avoid?), what is normal, and how to prevent or reduce future episodes. [32]<br>•Although parents suggested information should be free of jargon, they were keen that it should not omit important technical information that would facilitate their understanding. [41]<br>•Participants emphasized the importance of information being accessible.[41]<br>•The most popular suggestions were for an illustrated booklet with photographs and videos, which they thought would be more effective if shown in discussion with a professional. [41]
**Alternative evaluation**	**Evaluation criteria and process**	•Uncomplicated ARI (cough, cold, runny nose) is generally treated at home with commercial or traditional cough remedies. ... If fever is present, a wider variety of medicines are tried, including commercially available antipyretics (paracetamol), antibiotics (a widely available over-the-counter antibiotic is chlor- amphenicol syrup [Kemicitin]), and antihelminthics if the illness is perceived to be caused by worms. [29]<br>•Some believed that symptomatic treatments such as paracetamol or cough syrups had the same mode of action as antibiotics but that antibiotics were more potent so any illness would improve earlier with antibiotic treatment. [41]<br>•Antibiotics were frequently confused with other medicines that the respondents had used for pain. [2]<br>•Some villagers claimed to have no opinion over what constituted a good drug or over different qualities of drug. Those who did differentiate between better and worse drugs based their opinions the drugs' effectiveness in quickly curing the illness. [10]<br>•An efficacious drug was described as one which was powerful hence able to cure the disease and a non-efficacious drug as that which was weak and would not be able to cure the disease when used. [1]<br>•In the FGDs, a pattern was described where the treatment received corresponded to the degree of illness as perceived by the mothers. [42]<br>•The judgement about whether antibiotics were necessary or not thus appeared to be based on the perceived severity of the child's illness rather than its aetiology. [55]
	**Treatment regimens**	•Self-treatment prior to the OPD visit was common among patients. Most patients (43, 86%) had tried traditional therapies before their visit to the OPD. Common therapies that were mentioned included drinking Ayurvedic herbal preparations (n = 25, 50%), drinking boiled coriander (n = 20, 40%), drinking or eating ginger (n = 7, 14%), and steam inhalation (n = 17, 34%). In addition, 16 (32%) patients had used acetaminophen for their fever. [17]<br>•Monitoring and maintaining control of symptoms was seen as paramount to minimize discomfort and reduce the threat of harm.[44]<br>•Likewise, mothers, grandmothers or guardians accompanied others to seek advice only if symptoms were considered severe.[7]<br>•We identified 3 common expectations for the ARTI visit among all parent respondents, namely, a desire for diagnosis, reassurance that the child's symptoms did not indicate something serious, and strategies to resolve the child's symptoms. [41]<br>•Antibiotics were thought to be effective and fast in treating fever. [36]<br>• "I had to ask for it. I said nothing's working and I've done everything, and so I said I want it (antibiotics). Please can I get something stronger that you can prescribe?" [47]<br>•... we found that perceptions of the risks and benefits of antibiotics and intent to demand them did not vary according to race, ethnicity, or socioeconomic status. [37]<br>•Themes regarding beliefs and perceptions regarding antibiotic use did not appear to differ between higher and lower educated groups or between genders. [60]<br>•Nearly all mothers were familiar with the possibility of ''side effects'' with prescription medications, including antibiotics, such as rash or diarrhea. Overwhelmingly, these were considered to be common, relatively benign, and even an expected consequence of antibiotic use. [74]<br>• "I think it's fairly legitimate to weigh up the risks and benefits really in terms of understanding a course of antibiotics under those circumstances could prevent that child from becoming worse in some circumstances." [33]<br>•I don't know what they do technically but you know, they help your immune system fight diseases or something. [2]<br>•None of the parents discussed the difference between broad- and narrow-spectrum antibiotics.[37]<br>•All of the villagers thought that the drips would take effect faster and would provide more complete recovery than both oral drugs and injections, even for common cold. [10]<br>•Those participants who understood that antibiotics treat infection were often confused about bacterial and viral infections. [2]<br>•Participants did not express demanding an antibiotics prescription generally. Still, they thought that having a prescription for antibiotics decreased the uncertainty regarding the ARLTI, especially when they had been unwell for a long time.[6]<br>•A prescription for antibiotics seemed to represent more than a decision about treatment: it was a way of helping some parents to cope and an indication that their concerns had been taken seriously by the doctor. [41]<br>•A script for antibiotics was considered a necessary tangible benefit to compensate for the time and effort invested to visit the doctor and waiting in the waiting area. [47]<br>•Some reported that by the time they seek medical attention for URI symptoms, they expect an antibiotic in order to return to normal activities. [74]
**Antibiotics obtaining**	**Antibiotics obtaining**	***HCPs***<br>•Presentation to a clinic may require no more than reassurance that the condition is self-limiting and confirmation that the patient's approach to mitigate symptoms is appropriate. Most patients stated that they did not necessarily expect to be prescribed an antibiotic. [7]

		•At this point, most expected a prescription from their provider, and some reported simply calling their provider's office and requesting an antibiotic when they had URI symptoms that had been ''treated by an antibiotic in the past.'' [74]<br>•The main reasons given for this (antibiotics) expectation is antibiotics help them feel better, prevention of potential deterioration of illness, previous successful experience and investment of time and money to consult a doctor. [47]<br>•Their reasons for demanding antibiotics for a cold or flu varied between the groups. However, there was a consensus that the individual was in the best position to decide if an antibiotic is needed for themselves or their children, hence the expectation and sometimes demand for antibiotics. [47]<br>•Among some of the older parents, or those who remembered treatments from their own childhoods, there was a general sense that antibiotics are now prescribed less frequently than in the past....One parent stated that current pressures on health care costs might be a reason that clinicians prescribe less frequently [34]<br>•Some individuals mentioned that they would ask for antibiotics if the infection got into their chest or if their symptoms were interrupting their daily activities, especially for their children. However, they stated that at the end they would accept the doctors' opinions even if no antibiotics were prescribed. [94]<br>•When a provider does not prescribe an antibiotic, patients want them to clearly explain why they do not need an antibiotic to treat their illness. Patients suggested that this will build trust with their providers. [50]<br>• "You know, although I trust my doctor, they do make mistakes, and sometimes you have to push and advocate." "They did give me one [antibiotic for otitis] here because I made it a point where if I have to come back, it's another $20." [34]<br>•Several patients said that if they were not prescribed medications, then they would seek care from another provider until they received a prescription: "No, I will not accept it [if medications are not prescribed]. [17]<br>***Pharmacy (without prescription)***<br>•It was quite common for villagers to go to the pharmacy to buy antibiotics in case of headache, fever, sore throat and cough. [10]<br>•"A few times they have given me some pills [antibiotics] for something, if it has happened again after some time, then instead of coming, I had taken the medication directly" [6]<br>•Rationales for not consulting a physician given by patients without a prescription included previous positive experiences with specific antibiotics and that their choice of antibiotics at the time was based either on their own experience or on advice from a colleague or family member. [72]<br>•Some villagers were even surprised to hear from the researcher that antibiotics should not be available in the pharmacy without a prescription. [10]<br>•In the cases presented by the pharmacists, most often the patient had chosen the antibiotics themselves. "…he said 'I know what I have and what I should use'." (Pharmacist 4). The patients without a prescription also reported that they chose the medicine themselves.... "I selected this antibiotic [amoxicillin] because I think it is stronger than ampicillin, based on my experience." [72]<br>•Use of drugs was also influenced by how much money one could spend and presence of the drug on the market. [1]<br>***Home leftover***<br>•One individual revealed that they used leftover antibiotics from previous episodes, because they believed that the anti-biotic helped to clear up their cold pretty quick, even though they knew that they were not supposed to do that. [94]<br>• "A few times they have given me some pills [antibiotics] for something, if it has happened again after some time, then instead of coming, I had taken the medication directly" (P19, man, 48 years old). [7]
**Antibiotic consumption**	**Antibiotic consumption**	•For any medication, if participants felt that they were not getting any benefits from taking it or they felt recovered, they would stop using it [6]<br>• Thridly, there is the dynamic character of the use of antibiotics. Someone who refuses antibiotics today, may ask for them tomorrow or vice versa. Lay people are constantly weighing up the pros and cons. [30]<br>• "Actually, I started with one capsule three times a day, but my throat pain became more severe, so I increased the dosage to two capsules three times a day to get faster results." [72]<br>•Mothers strongly believed that medicines were not to be administered after the symptoms such as cough or fever had gone, otherwise there would a danger of overdosage....70% of caretakers considered antibiotics dangerous if taken for too long, too frequently, or after the symptoms had disappeared, and prolonged use of the drugs could cause deafness and abnormalities. [89]<br>•Most also agreed that information about ADEs would lead to increased vigilance on their part to ensure antibiotics were only used when needed but would not interfere with adherence to antibiotic prescriptions. [74]<br>•Ideas of what constituted "too long" a period to be receiving antibiotics varied from caretaker to caretaker, although typically at some time from 4-7 days, and most women relied on signs of regaining health in the sick child in order to vary dosage or cease administration. [89]<br>•For example, those who expressed a negative attitude toward medicines and were dissatisfied with doctors who overprescribe antibiotics tended to use lower doses or took antibiotics for a shorter duration, to eliminate the feeling of the guilt of not adhering to doctor's treatment advice. [93]<br>•Patients often do not understand how to take antibiotics as they are prescribed or the importance of taking antibiotics as they are prescribed. [50]
**Post-consumption evaluation**	**Enhanced misbelief of antibiotics as a strong medicine for URTIs**	•The people who chose to "nurse their illness" believed that their way of coping was well considered and that they made the right decision by not calling a doctor. But the same applies to the people who took medicines; they too recovered after taking medicines for a short period and hence also thought their decision was well considered. Besides, the diagnosis by the physician and the prescribed medicines often seemed to justify their choice. [30]<br>•Although some physicians and patients are aware that not every complaint needs to be treated by medicines, many Flemish are accustomed to leaving the consulting room with some medicine or other. This has given rise to a certain pattern of expectations, which is often reinforced by the prescription habits of the Flemish physicians. Besides, pressure on physicians from patients can be much stronger in Flanders. This reflex that "illness = seeing a doctor = medicines" leads to a high consumption of medicines, e.g. antibiotics. This leads to another vicious circle where the physician prescribes a medicine because he thinks patient expects it, and the patient expects a prescription because he is used to getting one. [30]
	**Acceptance of side effects**	•Across all network types there was an awareness that AMR is a public health issue caused by over use/ misuse of antibiotics and this awareness had influence over health seeking behavior. [5]<br>•No one in the group knew what antibiotic resistance was but they all had heard of the term before the focus group. [47]

•Parents who had heard of the term were quick to point out that antibiotic resistance occurs due to inappropriate use. [8]
•Most parents perceived that the body becomes "immune" to antibiotics, but only a few (n = 7 [6.4%]) parents mentioned resistant organisms. [37]
•No, there is no side effect, but there is resistance if you take too much (antibiotics). If you always take one kind of xiaoyanyao (antibiotics) and later it does not work, you can let the doctor change it for another one, and then that will work. [10]
•Parents appeared to have a sense of optimism about how antibiotic resistance was likely to affect their family. Most perceived their own family at low risk because they considered themselves to be low users of antibiotics. [8]
•Yet, some parents thought that the implications of antibiotic resistance, although a possibility, were a problem for the future and found them difficult to relate to. They thought that society would identify different ways of treating infections (e.g. discovery of new drugs). [8]
• "I think sometimes people, other parents, may be a little neurotic and worried and you know 'give [me] an antibiotic now!' And it might have been worth it to wait it out a little bit, but when parents take their kids to the doctor's, they expect something. You know, this isn't in my case, but I just know people who are like this, and if you come and you don't get anything, then you're like 'well, why did I bother?'" [37]
•Some perceived themselves as low users of antibiotics by using antibiotics 'only when needed' thereby reducing antibiotic resistance. Other parents were unsure as to how they could reduce antibiotic resistance themselves as the problem was part of a 'much bigger' picture and that a collective public effort was needed to use fewer antibiotics. [8]
• "In future, if (antibiotics) doesn't help anymore. You need to take a stronger pill. But that's in the future; you have to focus on this point in time." [47]
•All the participants mentioned side effects as a consequence of taking antibiotics but this didn't deter them. [47]
•Nearly all mothers were familiar with the possibility of ''side effects'' with prescription medications, including antibiotics, such as rash or diarrhea. Overwhelmingly, these were considered to be common, relatively benign, and even an expected consequence of antibiotic use. [74]

Supplementary file S5 - Other irrational antibiotic use behaviors of the public for URTIs and their influencing factors

Study	Behaviors		Influencing factors			
	Measured outcomes	Prevalence	Capability	Opportunity	Motivation	Individual characteristic
**Overall antibiotic rational use behaviors**						
Borg, M.A (2011)	Antibiotic use for cold/flu/sore throat in the previous 12 months	11%-81% (based on countries)	- Higher knowledge of antibiotics and AMR (↓)	- National culture (△)		
El Khoury, G.,et al (2017)	Parents' good practice of antibiotic use (expectation, adherence, self-medication and storage)	N/A	- Higher knowledge of antibiotic use (↑)<br>- Higher education (↑)<br>- Medical background (↑)	- Past experience (△)<br>- Information sources (↓)		
Lanyero, H (2020)	Antibiotic use for under 5 years children's URTIs	60.2%		- Living setting (peri-urban, ref: rural) (↑)<br>- Care-seeking places (a health facility) (↑)	- Presence of cough (↑)	
You, J.H. et al (2008)	Adults' good practice of antibiotic use (adherence and non-prescription use)	N/A				- Male (↓)
Hernández-Díaz, I et al (2019)	Parents' good practice of antibiotic use (self-medication and storage)	N/A	- Higher education (↑)	- More past experience of antibiotic use (↓)		- Younger age (↓)
Wilson, A.A. et al (1999)	Adults' antibiotic use for RTIs in 12 months	N/A	- Higher knowledge of antibiotic use (↑)	- Past experience of RTIs (↑)<br>- Information source (↑)	- Perceived antibiotic efficacy (↑)	- Having a chronic disease (↑)
Hassan, M.Z. et al (2020)	Mothers' antibiotic use for under-5 children's ARI in 2 weeks	38.70%		- Living setting (urban, ref: rural) (↓)<br>- Drug sources (△)		- Poor nutrition of Children (↓)
Bianco, A. et al(2020)	Adults' antibiotic use for common cold/fever	23.60%	- Higher knowledge of antibiotics and AMR (↓)- Higher education (↓)	- Past experience of antibiotic use (↑)		- Male (↑)

Gu, J., et al. et al	Adults' antibiotic use for different URTIs symptoms	24.0% - 41.9% (based on symptoms)	- Family member's medical background (only for antibiotic use for sore throat, congested nose with headache and clear sputum) (↑)<br>- Higher awareness of antibiotics (only for antibiotic use for sore throat, congested nose with headache and clear sputum) (↓)	- Living setting (Urban, ref: rural) (only for antibiotic use for sore throat, congested nose with headache and clear sputum) (↑)	- Younger age (only for antibiotic use for sore throat, congested nose with headache and clear sputum) (↓)<br>- Normal/good health status (only for antibiotic use for sore throat, congested nose with headache and clear sputum) (↓)
Larsson, M. et al	Caregivers' antibiotic use for children's RTIs in 4 weeks	75.00%			
**Antibiotic storage**					
El Khoury, G.,et al (2017)	Parents' antibiotic storage	6.70%			
Hernández-Díaz, I. et al (2019)	Parents' antibiotic storage	15.50%			
Parimi, N. et al (2004)	Caregivers' antibiotic storage	24.50%	- Limited knowledge (↓)		
**Antibiotic treatment postponing behavior**					
Rönnerstrand, B. et al (2015)	Willingness to postpone antibiotic treatment for RTIs (Days, Mean±SD)	4.03±2.13		- Perceived others behaviors to postpone antibiotic treatment (△)	- Generalized trust of others (↑)<br>- Poor health (↓)
**Future antibiotic expectation after use of prescribed antibiotics**					
Emslie, M.J. et al (2003)	Adults' future antibiotic expectation who have used prescribed antibiotics	74.40%			

D. Osborne and H. Sinclair (2006)	Patients' future antibiotic expectation who have used prescribed antibiotics	81.80%
